# A Clinical Semantic and Radiomics Nomogram for Predicting Brain Invasion in WHO Grade II Meningioma Based on Tumor and Tumor-to-Brain Interface Features

**DOI:** 10.3389/fonc.2021.752158

**Published:** 2021-10-22

**Authors:** Ning Li, Yan Mo, Chencui Huang, Kai Han, Mengna He, Xiaolan Wang, Jiaqi Wen, Siyu Yang, Haoting Wu, Fei Dong, Fenglei Sun, Yiming Li, Yizhou Yu, Minming Zhang, Xiaojun Guan, Xiaojun Xu

**Affiliations:** ^1^ Department of Radiology, the Second Affiliated Hospital, Zhejiang University School of Medicine, Hangzhou, China; ^2^ Department of Radiology, Fuyang District First People’s Hospital, Hangzhou, China; ^3^ Deepwise AI Lab, Beijing Deepwise & League of PHD Technology Co., Ltd., Beijing, China

**Keywords:** atypical meningioma, brain invasion, magnetic resonance imaging, radiomics, semantic

## Abstract

**Background:**

Brain invasion in meningioma has independent associations with increased risks of tumor progression, lesion recurrence, and poor prognosis. Therefore, this study aimed to construct a model for predicting brain invasion in WHO grade II meningioma by using preoperative MRI.

**Methods:**

One hundred seventy-three patients with brain invasion and 111 patients without brain invasion were included. Three mainstream features, namely, traditional semantic features and radiomics features from tumor and tumor-to-brain interface regions, were acquired. Predictive models correspondingly constructed on each feature set or joint feature set were constructed.

**Results:**

Traditional semantic findings, e.g., peritumoral edema and other four features, had comparable performance in predicting brain invasion with each radiomics feature set. By taking advantage of semantic features and radiomics features from tumoral and tumor-to-brain interface regions, an integrated nomogram that quantifies the risk factor of each selected feature was constructed and had the best performance in predicting brain invasion (area under the curve values were 0.905 in the training set and 0.895 in the test set).

**Conclusions:**

This study provided a clinically available and promising approach to predict brain invasion in WHO grade II meningiomas by using preoperative MRI.

## 1 Introduction

Brain invasion becomes a stand-alone criterion for atypical grade II meningioma in the updated 2016 World Health Organization (WHO) Classification of Tumors of the CNS (1), because of its independent associations with increased risks of tumor progression, lesion recurrence, and poor prognosis (2–5). Therefore, the existence of brain invasion can significantly impact preoperative evaluation and decision-making. Regarding this rising clinical significance, the recognition of brain invasion for brain meningioma especially before clinical intervention is very important, but few biomarkers are routinely used in clinical practice.

As the only golden standard for the diagnosis of brain invasion in meningioma, histopathological examination is greatly dependent on the acquisition of peritumoral brain tissue, leading to a heterogeneous assessments of brain invasion (6). Alternatively, in the preoperative diagnosis/assessment, magnetic resonance imaging (MRI) is the most important technique for brain meningioma by taking advantage of its ultra-high tissue resolution and spatial resolution. Previous existing documents suggested that traditional MRI findings, like peri-tumoral edema, heterogeneous contrast enhancement, and irregular tumor shape, have values in predicting brain invasion (6, 7). However, the outcomes of these imaging signs are not widely supportive (8), which may be resulting from the limited and insufficient information they provided.

Radiomics can convert medical images into mineable high-dimensional quantitative data that may reflect underlying pathophysiology of the tumor (9). By employing radiomics, a number of studies reported the relevant values in grading and classifying brain meningiomas (10–13), while only several documents related it to predict brain invasion in meningioma. Zhang et al. demonstrated that some radiomics features within tumor and sex jointly reached the best performance in predicting brain invasion (14). Joo et al. constructively suggested that the radiomics features from the tumor-to-brain interface region could help predict brain invasion in meningioma (15). Therefore, this couple of studies leads an important role in introducing radiomics to assess the risk of brain invasion in meningioma. However, it is worth noting that 1) both studies merely arbitrarily extracted radiomics features from the tumor region or tumor-to-brain interface region and (2) WHO grade I meningiomas occupied the majority of the training dataset, which might bring pathological bias in model construction (14, 15). Therefore, since grade I meningioma with brain invasion has been assigned to WHO grade II (1), it deserves to predict brain invasion in high grade meningioma (WHO grade II) by integrating the value of radiomics features in tumor and tumor-to-brain interface regions, as well as the traditional radiological findings (semantic features).

In the present study, three mainstream features, namely, radiomics features from the tumor region, radiomics features from the tumor-to-brain interface region, and semantic features, were subsequently extracted from each meningioma. Feature selection and model construction were conducted step by step, and the value of each selected feature was estimated. Finally, an integrated nomogram constructed on the selected features was built to comprehensively estimate the risk points as a composite predictor for brain invasion in meningioma.

## 2 Materials and Methods

This retrospective study was approved by the Medical Ethics Committee of the Second Affiliated Hospital of Zhejiang University School of Medicine. The written informed consent from the patients was waived. All the methods were carried out in accordance with relevant guidelines and regulations.

### 2.1 Subjects

**Figure 1:**
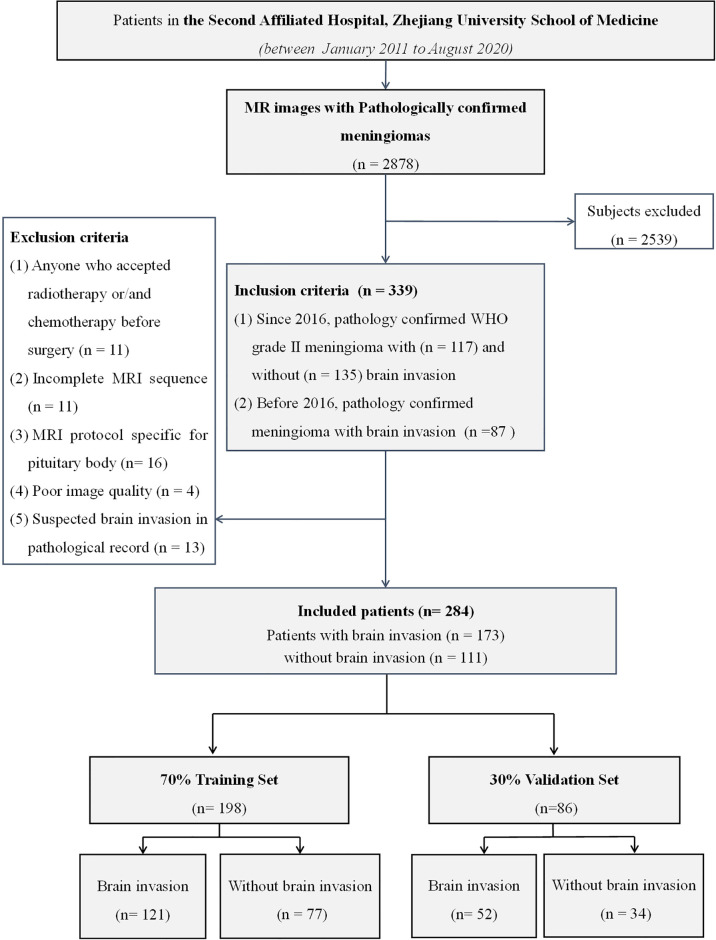
The flowchart of data inclusion and exclusion.

Initially, 2,878 meningioma patients with pathological confirmation from January 2011 to August 2020 were screened. In the 2016 WHO Classification of Tumors of the CNS, a significant revision for meningioma was that the presence of brain invasion in a WHO grade I meningioma is assigned to WHO grade II (1). Thus, in consideration of this update, a total of 339 patients were included according to the following inclusion criteria: 1) since 2016, WHO grade II meningioma with (*N* = 117) and without (*N* = 135) brain invasion should have histopathological evidence; and 2) before 2016, because histopathological assessment of brain invasion was not a regular guideline for grading meningioma, only meningioma with brain invasion (*N* = 87) was histopathologically confirmed and included. Then, 55 patients were further excluded according to the exclusion criteria shown in **Figure 1**. Finally, 173 meningiomas with brain invasion and 111 meningiomas without brain invasion were recruited.

### 2.2 Image Acquisition

All the MRI examinations were completed 1 week before the operation in the Second Affiliated Hospital of Zhejiang University School of Medicine. All the images (T1-weighted, T2-weighted, and enhanced T1-weighted imaging) were acquired using clinical scanning protocols in eight MRI scanners (3.0 T scanners, e.g., GE Discovery MR 750, GE Discovery MR 750W, GE Signal HDxt, and United Imaging MRI 790; 1.5 T scanners, e.g., Siemens Magnetom Aera, Siemens Magnetom Avento, Siemens Magnetom Sonata, and GE Signal HDxt).

### 2.3 Clinical Semantic Assessment

Two neuroradiologists with 5 years of experience who were blinded to the clinical and pathological information of the patients evaluated the clinical semantic features for each meningioma. When an inconsistency occurred, the result will be rechecked by a senior neuroradiologist with 30 years of experience. Semantic features including radiological findings and demographic information were recorded (10, 11): 1) tumor location: anterior/middle/posterior cranial fossa, sphenoid crest, saddle tubercle, lateral/midline convexity, tentorium cerebelli, ventricle, other; 2) number of tumors: single or multiple; 3) the largest diameter of the tumor; 4) short diameter perpendicular to the maximum length diameter; 5) T1 signal intensity; 6) T2 signal intensity; 7) degree of contrast enhancement on gadolinium-enhanced T1 imaging; 8) intratumoral heterogeneity after enhancement; 9) tumor margin; 10) peritumoral edema; 11) cystic or necrosis; 12) bone invasion; 13) hyperostosis; 14) dural tail; 15) venous sinus invasion; 16) CSF cleft sign; 17) arterial narrowing; 18) sunburst; 19) age; and 20) sex.

### 2.4 Radiomics Modeling

#### 2.4.1 Semi-Automatic Region of Interest Segmentation

**Figure 2:**
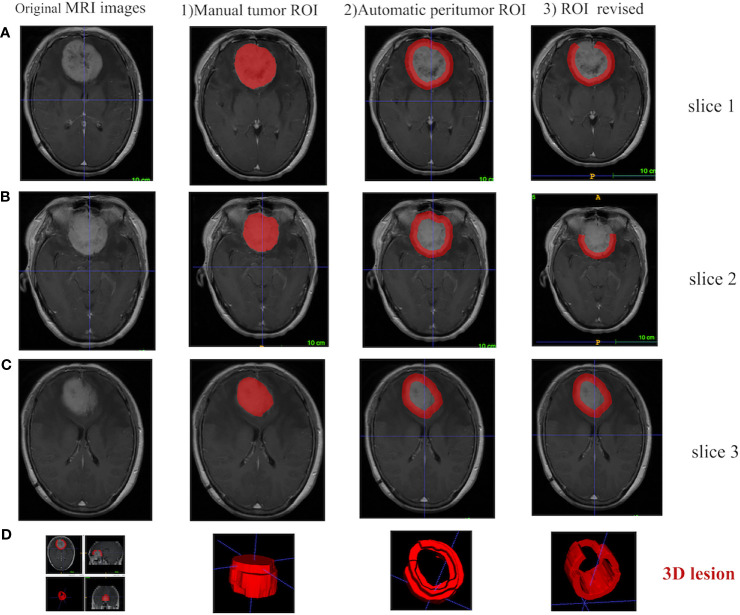
Different ROI segmentation conditions are displayed in 2D and 3D in ITK-SNAP software, including the original image, the manually segmented tumoral ROI, and the semi-automatically segmented tumor-to-brain interface ROI. **(A)** Tumor located in anterior cranial fossa with overlap of non-brain tissues (i.e., bone) after 5 mm expansion, which is manually revised to only keep tumor-to-brain interface. **(B)** The same tumor with overlap of non-brain tissues (i.e., postorbital tissues) after 5 mm expansion, which is manually revised to only keep tumor-to-brain interface. **(C)** The same tumor without any overlap of non-brain tissues after 5 mm expansion. **(D)** 3D visualization. ROI, region of interest.

For every meningioma lesion, manual segmentation was conducted to extract the tumor region, while a semi-automatic segmentation was used to acquire the tumor-to-brain interface region (**Figure 2**). The details were shown below:

1) Manual segmentation of the tumor region [region of interest (ROI)]. Two radiologists with about 5 years of clinical experience manually segmented the tumor ROI along the sharp tumor margin in the axial enhanced T1-weighted images in a slice-by-slice way. Before manual segmentation, these two radiologists were trained by a neuroradiologist with 30 years of experience, and then both of them blinded to the patient information manually segmented 40 randomly selected tumors. DICE similarity coefficient was calculated to test the interoperator agreement (16, 17). As a result, the DICE similarity coefficient was 0.914 ± 0.035, indicating an excellent agreement.

2) Automatic segmentation of tumor-to-brain interface ROI. Based on the outer edge of the tumor region segmented in the first step, the 5 mm in the spatial scale was firstly converted to the pixel scale in the image, and then the morphology operations of image expansion and corrosion (Python, Skimage.Morphology) (18) were carried out to automatically segment the tumor-to-brain interface ROI. The initial region was formed by the annular region with the outer boundary of the tumor and the amplification boundary as the inner and outer boundary.

3) Final review and revision for the tumor-to-brain interface region. The initial tumor-to-brain interface region was reviewed layer by layer by the neuroradiologist. If the expansion boundary included non-interested brain/non-brain regions, manual correction was carried out; if no correction was needed, automatic segmentation was retained.

#### 2.4.2 Image Preprocessing and Radiomics Feature Extraction

**Figure 3:**
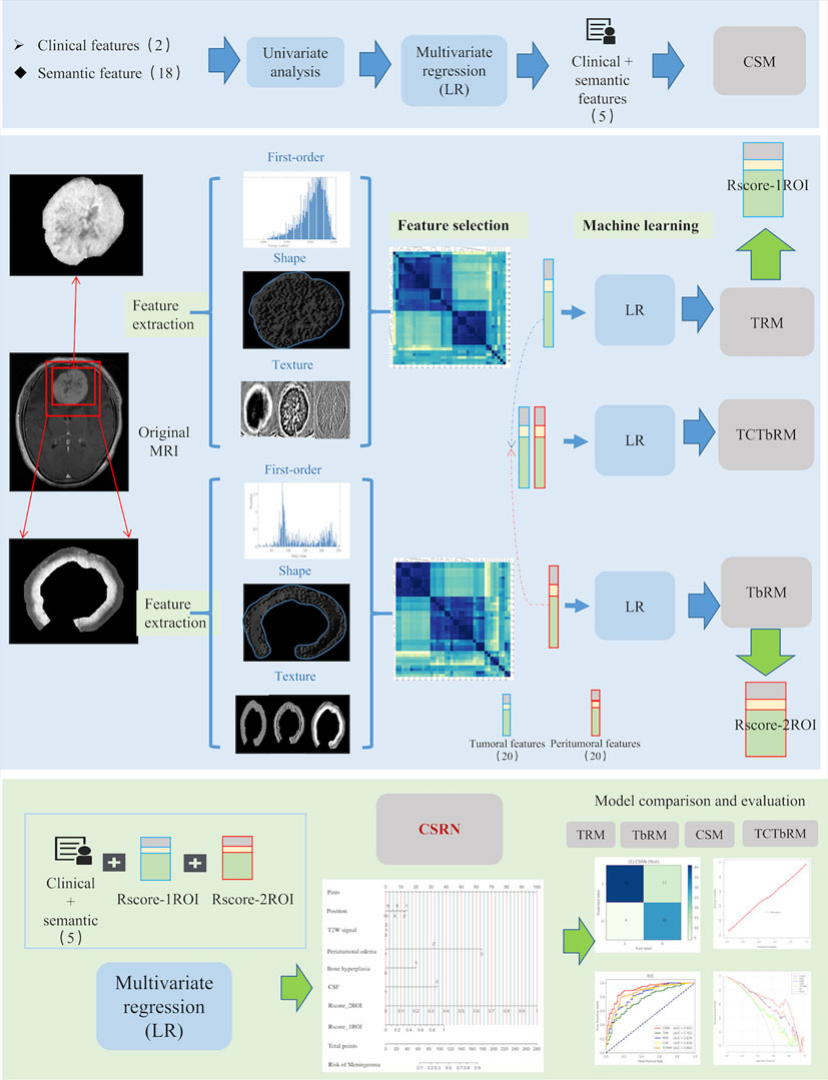
Workflow of this study, which mainly composed of six steps: ROI segmentation, image preprocessing, feature extraction, feature selection, model building, and model comparative evaluation. ROI, region of interest.

The original MRI images and the corresponding annotation files were upload to the Deepwise multimodal research platform (https://keyan.deepwise.com, V1.6.2) for radiomics feature quantification, feature engineering on the volume map of the semi-automatically labeled two-dimensional ROI. The complete process of this study is shown in **Figure 3**, which is mainly composed of six steps: ROI segmentation, image preprocessing, feature extraction, feature selection, model building, and model evaluation.

Firstly, in the image preprocessing, *Z*-score normalization was used to process the images with a normalize scale of 100 (19), and the B-spline interpolation sampling method was used to resample MRI images with different resolutions to the same resolution [1,1,1] (20). Then, eight different image transforms (https://pyradiomics.readthedocs.io/en/latest/radiomics.html#module-radiomics.imageoperations), such as high-pass wavelet filter, low-pass wavelet filter, Laplace, gradient, and Gaussian transform, were used to obtain more pixel-level high-throughput image features. Secondly, based on the original and transformed images, we extracted and quantified the radiomics features of tumor and peritumor ROIs, respectively, which included three categories: first-order, shapes, and texture features (21). The three described global information such as gray mean value and variance, local information such as shape and edge of ROI, and mutual information between pixels inside ROI and neighborhood, respectively. Texture features mainly include the GLCM (gray level co-occurrence matrix), GLRLM (gray level run length matrix), GLSZM (gray level size zone matrix), GLDM (gray level dependence matrix), and NGLD (neighboring gray level dependence matrix) (https://pyradiomics.readthedocs.io/en/latest/features.html). See **Supplement Material 1** for specific features.

Finally, a total of 1,763 radiomics features were extracted and normalized for each ROI in our study. *Z*-score normalization was used to eliminate the influence of feature dimensions and speed up the solution of the gradient descent algorithm, *Z* = (*X* − mean)/SD.

#### 2.4.3 Features Selection of Radiomics and Semantic Features

##### 2.4.3.1 Selection of Radiomics Features

It consisted of two stages: first, interobserver interclass coefficient (ICC) analysis and correlation analysis were used (22, 23). ICC analysis was used to exclude features with interobserver instability (ICC coefficient < 0.9), and correlation analysis between features was used to exclude features with high correlation (Pearson correlation coefficient > 0.7) and retain low correlation (Pearson correlation coefficient < 0.7). Secondly, the *F*-hypothesis test (ANOVA, *F*-test of homogeneity of variance) (https://statisticsbyjim.com/anova/f-tests-anova/) was used for further feature selection. The *F*-test looked for the linear relationship between the two data groups and returned two statistics of *F*-value and *P*-value. We retain the features that were significantly correlated with the true label (*P*-value < 0.01) and delete those without significantly linear correlation (*P*-value > 0.01) (https://scikit-learn.org/stable/modules/feature_selection.html).

##### 2.4.3.2 Selection of Semantic Features

Statistical tests, univariate and multivariate analyses, and stepping-regression methods were used to select semantic features which were associated with brain invasion of meningioma.

#### 2.4.4 CSRN Construction

The significant semantic and radiomics features were selected as the independent variables, while the meningioma invasion was taken as the dependent variable. The logistic regression (LR) was used to establish a multivariate regression model for predicting brain invasion for meningioma.

We developed five models, namely, 1) tumoral radiomics model (TRM), 2) tumor-to-brain interface radiomics model (TbRM), 3) clinical semantic model (CSM), 4) tumor combined tumor-to-brain interface radiomics model (TCTbRM), and 5) clinical semantic and radiomics nomogram (CSRN).

LR is a traditional machine learning binary classifier, which is often used to analyze the risk factors of a certain disease and is suitable for predicting categorical variable (such as meningioma invasion and non-invasion events in this study) (24). This method could output a quantized non-linear model and probabilistic values (continuous variable).

The CSRN was established and evaluated as follows:

1) Model training. All patients were divided into training set and test set in a ratio of 7:3, and it was iterated for 2,000 times to get a stable result. Considering the AUC performance of the training set and test set comprehensively, and following the fact that the number of modeling features accounted for 10%–20% of the total sample size to simplify the prediction model (25), we selected radiomics features, respectively, and examined their statistical differences between meningioma with and without brain invasion.

2) Calculation of radiomics scores. TRM and TbRM based on LR were constructed by selecting 20 significant tumor and 20 tumor-to-brain interface radiomics features, respectively, and the output probability scores of the combination of modeling features and weights were converted into radiomics score, Rad_score (Rscore_1ROI, Rscore_2ROI) (26).


$$\text{Rad}\_\text{score}=\frac{1}{1+\exp(\Sigma \;\beta_{\text{i}}*{\text{f}}_{\text{i}})}$$

f_i_ represents radiomics feature i, while ß_i_ represents the coefficient corresponding to this feature.

3) Quantitative representation of CSRN. With the inclusion of significant semantic features, Rscore_1ROI and Rscore_2ROI, a CSRN for predicting the meningioma invasion probability was established using multivariate LR (24). Thus, each factor and the predicted probability of brain invasion were described and calculated numerically.

4) Establishment of different models. Similarly, we extracted the features of single category and multiple categories, respectively, and established the remaining four models, namely, TRM, TbRM, CSM, and TCTbRM. See **Supplement Material 2** for details.

5) Comparison and evaluation among the models. The semantic features, tumoral radiomics features, and tumor-to-brain interface radiomics features involved in the modeling were discussed in detail for their application value in clinical scenarios, and the contribution and clinical significance of this study to predict the invasion of WHO grade II meningiomas were also discussed.

The ROC curve, the area under the ROC curve (AUC), accuracy, sensitivity, specificity, negative predictive value (NPV), and positive predictive value (PPV) indexes comprehensively described the performance of the five classifiers. Calibration curves were used to describe the predictive accuracy of CSRN, and decision curve analysis (DCA) was used to describe the clinical efficacy between the models. Feature heat maps were used to describe the correlations between radiomics features, and Python’s image processing package was used to visualize these features.

### 2.5 Statistical Analysis

SPSS 22.0 (released 2013; IBM SPSS Statistics for Windows, Version 22.0), R (https://www.rstudio.com), Python 4.0 (https://www.python.org/), and Deepwise DXAI Platform (https://dxonline.deepwise.com/) were used for statistical validation, analysis, and visualization. Mean and standard deviation (SD) were used to describe numerical variables. Two-independent sample *t*-test was used for the variables with normal distribution, while Wilcoxon test was used for skewed distribution. Frequency was used to describe categorical variables, chi-square test or corrected chi-square test was used for disordered variables, and Kruskal–Wallis *H* test was used for ordered variables. DeLong test was used to compare the ROC curves among the five models, and *Z*-test was used to compare the differences between AUC, accuracy, sensitivity, specificity, NPV, PPV, and other indicators. This study was a bilateral significance test, and a two-tailed *P <*0.05 was considered statistically significant.

## 3 Results

### 3.1 Demographic Information

A total of 284 patients with WHO grade II meningioma were enrolled, consisting of 173 patients with brain invasion and 111 patients without brain invasion. **Table 1** specifies the overall distribution of demographic information and semantic features.

**Table 1:** Demographic information of the 284 patients.

Index	Patients (*n* = 284)		
	Non-invasion group (*n* = 111)	Invasion group (*n* = 173)	*p*-value
**Age**	57.1 ± 12.3	56.6 ± 11.5	0.745^a^
**The largest diameter of the tumor**	42.8 ± 16.8	44.6 ± 14.5	0.358^a^
**Short diameter perpendicular to the maximum length diameter**	31 (24–39.5)	34 (25–41)	0.226^b^
**Sex**			0.843^c^
Female	68 (61.3%)	108 (62.4%)	
Male	43 (38.7%)	65 (37.6%)	
**Tumor location**			0.000**^c^
Anterior cranial fossa	7 (6.3%)	38 (22.0%)	
Middle cranial fossa	5 (4.5%)	3 (1.7%)	
Posterior cranial fossa	11 (9.9%)	3 (1.7%)	
Sphenoid crest	10 (9.0%)	14 (8.1%)	
Saddle tubercle	0 (0.0%)	4 (2.3%)	
Lateral convexity	44 (39.6%)	78 (45.1%)	
Midline convexity	24 (21.6%)	20 (11.6%)	
Tentorium cerebelli	7 (6.3%)	9 (5.2%)	
Ventricle	1 (0.9%)	4 (2.3%)	
Other	2 (1.8%)	0 (0.0%)	
**Number of tumors**			0.073^c^
Single	109 (98.2%)	162 (93.6%)	
Multiple	2 (1.8%)	11 (6.4%)	
**Cystic or necrosis**	59 (53.2%)	105 (60.7%)	0.209^c^
**Bone invasion**	47 (42.3%)	67 (38.7%)	0.544^c^
**Hyperostosis**	43 (38.7%)	96 (55.5%)	0.006**^c^
**Venous sinus invasion**	18 (16.2%)	30 (17.3%)	0.805^c^
**Dural tail**	100 (90.1%)	148 (85.5%)	0.262^c^
**CSF cleft sign**	97 (87.4%)	126 (72.8%)	0.004**^c^
**Arterial narrowing**	4 (3.6%)	6 (3.5%)	1.000^c^
**Sunburst**	1 (0.9%)	3 (1.7%)	0.948^c^
**T1**			0.200^d^
Hyperintense	6 (5.4%)	2 (1.2%)	
Isointense	73 (65.8%)	113 (65.3%)	
Hypointense	32 (28.8%)	58 (33.5%)	
**T2**			0.029^d^*
Hyperintense	49 (44.1%)	68 (39.3%)	
Isointense	61 (55.0%)	91 (52.6%)	
Isointense	1 (0.9%)	14 (8.1%)	
**T1+C (degree of CE)**			0.325^d^
Mild	15 (13.5%)	31 (17.9%)	
Marked	96 (86.5%)	142 (82.1%)	
**Intratumoral heterogeneity**			0.689^d^
Uniformly	41 (36.9%)	68 (39.3%)	
Uneven	70 (63.1%)	105 (60.7%)	
**Tumor margins**			0.572^d^
Clear	24 (21.6%)	32 (18.5%)	
Unclear	55 (49.5%)	88 (50.9%)	
Indistinct	32 (28.8%)	53 (30.6%)	
**Peritumoral edema**			0.000**^d^
None	38 (34.2%)	10 (5.8%)	
Mild	55 (49.5%)	101 (58.4%)	
Marked	18 (16.2%)	62 (35.8%)	

^a^Two sample t-test. ^b^Wilcoxon test. ^c^Chi-square test. ^d^Kruskal–Wallis H test. *p < 0.05, **p < 0.01.

No significant difference in age, the largest diameter of the tumor, and the short diameter perpendicular to the maximum length diameter was observed between meningiomas with and without brain invasion (*P* > 0.05), while significant differences in tumor location, hyperostosis, CSF cleft sign, T2-weighted signal, and peritumoral edema were observed between two groups (*P* < 0.05), suggesting that meningiomas with brain invasion had higher frequency in the location of anterior cranial fossa but lower frequency in midline convexity; higher frequencies of hyperostosis, hypointense T2-weighted signal, and peritumoral edema; and lower frequency of CSF cleft sign in comparison with meningioma without brain invasion (**Table 2**).

**Table 2:** Demographic information of meningioma patients in the training set and test set.

Index	Training set (*n* = 198)			Test set (*n* = 86)			*p*-value
	Non-invasion group (*n* = 77)	Invasion group (*n* = 121)	*p*-value	Non-invasion group (*n* = 34)	Invasion group (*n* = 52)	*p*-value	
**Age**	57.1 ± 11.7			56.2 ± 12.2			0.564^a^
**The largest diameter of the tumor**	42.2 ± 16.4	45.6 ± 14.3	0.132^a^	44.3 ± 17.7	42.3 ± 14.8	0.571^a^	0.542^a^
**Short diameter perpendicular to the maximum length diameter**	31 (24–39)	34.8 (26–41)	0.092^b^	34.5 (24.3–41.3)	34 (23.5–40.1)	0.477^b^	0.899^b^
**Sex**			0.934			0.803^c^	0.324^c^
Female	46 (59.7%)	73 (60.3%)		22 (64.7%)	35 (67.3%)		
Male	31 (40.3%)	48 (39.7%)		12 (35.3%)	17 (32.7%)		
**Tumor location**			0.010**			0.053^c^	0.769^c^
Anterior cranial fossa	5 (6.5%)	27 (22.3%)		2 (5.9%)	11 (21.2%)		
Middle cranial fossa	4 (5.2%)	3 (2.5%)		1 (2.9%)	0 (0.0%)		
Posterior cranial fossa	7 (9.1%)	2 (1.7%)		4 (11.8%)	1 (1.9%)		
Sphenoid crest	8 (10.4%)	11 (9.1%)		2 (5.9%)	3 (5.8%)		
Saddle tubercle	0 (0.0%)	2 (1.7%)		0 (0.0%)	2 (3.8%)		
Lateral convexity	32 (41.6%)	52 (43.0%)		12 (35.3%)	26 (50.0%)		
Midline convexity	15 (19.5%)	16 (13.2%)		9 (26.5%)	4 (7.7%)		
Tentorium cerebelli	4 (5.2%)	5 (4.1%)		3 (8.8%)	4 (7.7%)		
Ventricle	0 (0.0%)	3 (2.5%)		1 (2.9%)	1 (1.9%)		
Other	2 (2.6%)	0 (0.0%)		0 (0.0%)	0 (0.0%)		
**Number of tumors**			0.162			0.932^c^	1.000^c^
Single	76 (98.7%)	113 (93.4%)		33 (97.1%)	49 (94.2%)		
Multiple	1 (1.3%)	8 (6.6%)		1 (2.9%)	3 (5.8%)		
**Cystic or necrosis**	42 (54.5%)	74 (61.2%)	0.357	17 (50.0%)	31 (59.6%)	0.380^c^	0.664^c^
**Bone invasion**	31 (40.3%)	47 (38.8%)	0.842	16 (47.1%)	20 (38.5%)	0.429^c^	0.697^c^
**Hyperostosis**	30 (39.0%)	68 (56.2%)	0.018*	13 (38.2%)	28 (53.8%)	0.156^c^	0.778^c^
**Venous sinus invasion**	10 (13.0%)	22 (18.2%)	0.333	8 (23.5%)	8 (15.4%)	0.343^c^	0.614^c^
**Dural tail**	68 (88.3%)	106 (87.6%)	0.882	32 (94.1%)	42 (80.8%)	0.153^c^	0.670^c^
**CSF cleft sign**	66 (85.7%)	93 (76.9%)	0.127	31 (91.2%)	33 (63.5%)	0.004**^c^	0.267^c^
**Arterial narrowing**	2 (2.6%)	6 (5.0%)	0.651	2 (5.9%)	0 (0.0%)	0.299^c^	0.711^c^
**Sunburst**	0 (0.0%)	1 (0.8%)	1.000	1 (2.9%)	2 (3.8%)	1.000^c^	0.158^c^
**T1**			0.671			0.089^d^	0.223^d^
Hyperintense	4 (5.2%)	1 (0.8%)		2 (5.9%)	1 (1.9%)		
Isointense	47 (61.0%)	79 (65.3%)		26 (76.5%)	34 (65.4%)		
Hypointense	26 (33.8%)	41 (33.9%)		6 (17.6%)	17 (32.7%)		
**T2**			0.069			0.897^d^	0.749^d^
Hyperintense	37 (48.1%)	45 (37.2%)		12 (35.3%)	23 (44.2%)		
Isointense	39 (50.6%)	68 (56.2%)		22 (64.7%)	23 (44.2%)		
Hypointense	1 (1.3%)	8 (6.6%)		0 (0.0%)	6 (11.5%)		
**T1+C (degree of CE)**			0.257			1.000^d^	0.168^d^
Mild	11 (14.3%)	25 (20.7%)		4 (11.8%)	6 (11.5%)		
Marked	66 (85.7%)	96 (79.3%)		30 (88.2%)	46 (88.5%)		
**Intratumoral heterogeneity**			0.987			0.451^d^	0.789^d^
Uniform	30 (39.0%)	47 (38.8%)		11 (32.4%)	21 (40.4%)		
Uneven	47 (61.0%)	74 (61.2%)		23 (67.6%)	31 (59.6%)		
**Tumor margins**			0.514			0.961^d^	0.830^d^
Clear	19 (24.7%)	21 (17.4%)		5 (14.7%)	11 (21.2%)		
Unclear	35 (45.5%)	64 (52.9%)		20 (58.8%)	24 (46.2%)		
Indistinct	23 (29.9%)	36 (29.8%)		9 (26.5%)	17 (32.7%)		
**Peritumoral edema**			0.000**			0.000**^d^	0.587^d^
None	27 (35.1%)	6 (5.0%)		11 (32.4%)	4 (7.7%)		
Mild	35 (45.5%)	72 (59.5%)		20 (58.8%)	29 (55.8%)		
Marked	15 (19.5%)	43 (35.5%)		3 (8.8%)	19 (36.5%)		

a Two-sample t-test.

b Wilcoxon test.

c Chi-square analysis.

d Kruskal–Wallis H test. *p < 0.05, **p < 0.01.

### 3.2 Radiomics Features Selection and Significance Analysis

**Figure 4:**
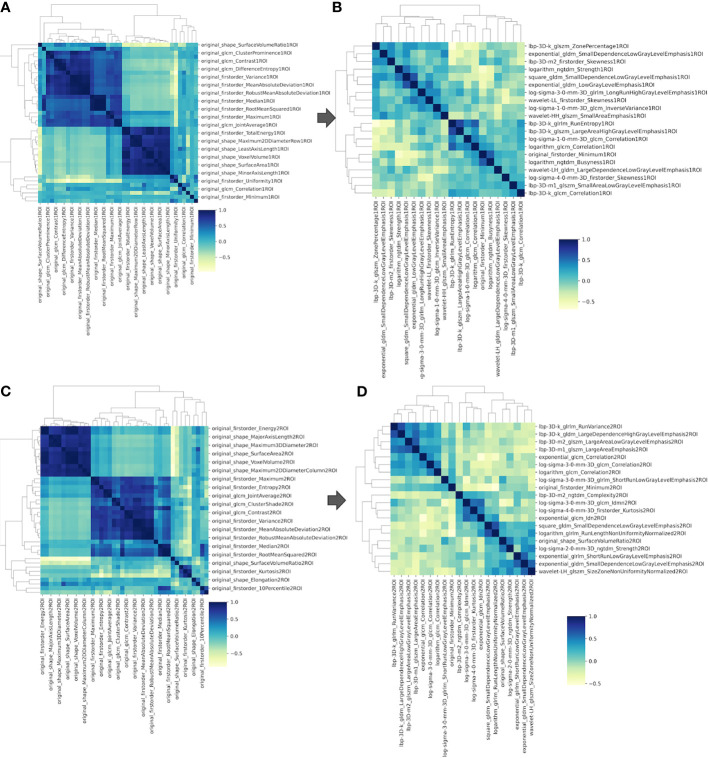
The Pearson correlation heat maps of radiomics features. **(A)** Sixty of the original 1,740 radiomics features of tumoral ROI; **(B)** 20 selected radiomics features of tumoral ROI; **(C)** 60 of the original 1,740 radiomics features of tumor-to-brain interface ROI; **(D)** 20 selected radiomics features of tumor-to-brain interface ROI. ROI, region of interest.

**Figure 5:**
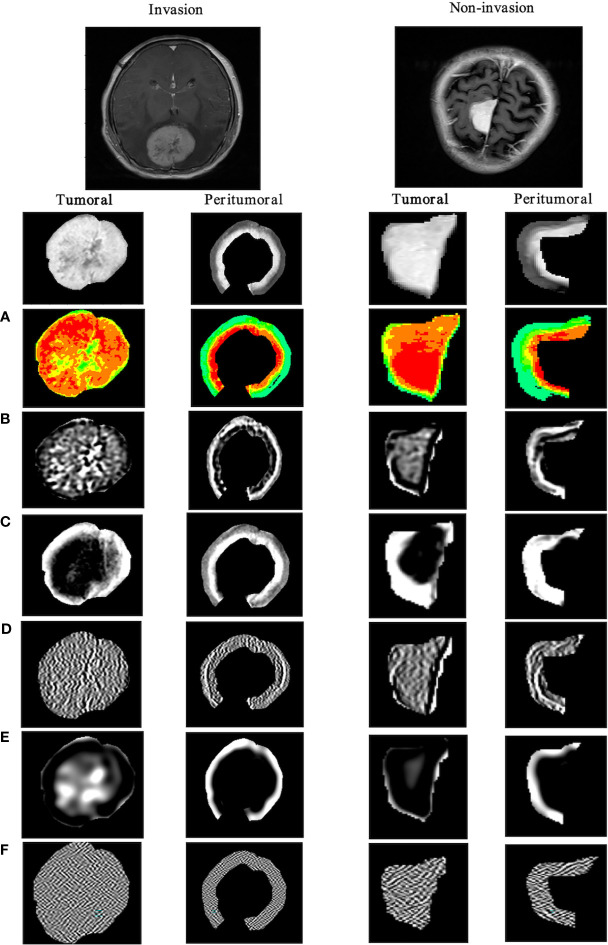
Visualization of tumoral and tumor-to-brain interface significant radiomics features of brain invasion and non-invasion in patients with meningioma. The results show the differences between two ROIs in the high-throughput radiomics features. In meningioma with brain invasion, the signal in the tumor is more dense, and the texture signal intensity around the 5-mm tumor is higher, that is, the information complexity is higher. **(A)** Original_firstorder (pseudo-color image); **(B)** wavelet-LLH_gldm; **(C)** log-sigma-1-0-mm-_glcm; **(D)** lbp-m2_ngtdm; **(E)** log-sigma-3-0-mm-_glrlm; **(F)** wavelet-HHL_glszm. ROI, region of interest.

A total of 1,740 tumoral and 1,740 tumor-to-brain interface radiomics features were extracted. After ICC analysis and correlation analysis, 20 tumoral and 20 tumor-to-brain interface features were selected using *F*-test and LR methods. The Pearson correlation heat maps of the original features and the selected features were respectively shown in **Figure 4**, and it could be clearly seen that the selected 20 features had low correlation in pairs, which reduced the feature redundancy. The radiomics feature distribution of randomly selected meningioma cases with and without brain invasion for each is shown in **Figure 5**. All the selected radiomics features are summarized in **Table 3** and ranked according to their classification contributions (absolute value of weights). Among 40 radiomics features, texture features *vs.* first-order features *vs.* shape features = 1.8162 *vs* 0.2743 *vs.* 0.0643 (about 28:4:1, the ratio of absolute value to the sum).

**Table 3:** Statistics of all the selected radiomics features.

Features	Weights	Mean	Standard deviation	*p*-value	Test	Feature source	Sort	Image
LoG-sigma-3-0-mm-3D_glrlm_ShortRunLowGrayLevelEmphasis2ROI	0.1468	0.0069	0.0058	0.001*	W	Tumor-to-brain interface ROI	Texture	LoG
LBP-3D-m2_ngtdm_Complexity2ROI	−0.1455	3.8204	1.2285	0.004*	W	Tumor-to-brain interface ROI	Texture	LBP
exponential_gldm_SmallDependenceLowGrayLevelEmphasis2ROI	−0.1226	0.0199	0.0024	0.000*	*t*	Tumor-to-brain interface ROI	Texture	Exponential
square_gldm_SmallDependenceLowGrayLevelEmphasis2ROI	−0.1015	0.0228	0.0057	0.000*	W	Tumor-to-brain interface ROI	Texture	Square
logarithm_ngtdm_Busyness1ROI	0.0942	0.0975	0.1218	0.000*	W	Tumor ROI	Texture	LoG
LoG-sigma-4-0-mm-3D_firstorder_Kurtosis2ROI	−0.0935	2.9566	0.8304	0.000*	W	Tumor-to-brain interface ROI	First-order	LoG
LoG-sigma-3-0-mm-3D_glcm_Correlation2ROI	0.0923	0.9341	0.0101	0.000*	W	Tumor-to-brain interface ROI	Texture	LoG
LoG-sigma-3-0-mm-3D_glcm_Idmn2ROI	−0.0921	0.996	0.0016	0.007*	*t*	Tumor-to-brain interface ROI	Texture	LoG
logarithm_ngtdm_Strength1ROI	−0.0882	26.2279	32.0014	0.000*	W	Tumor ROI	Texture	LoG
LoG-sigma-1-0-mm-3D_glcm_InverseVariance1ROI	0.0828	0.4085	0.0516	0.000*	W	Tumor ROI	Texture	LoG
LoG-sigma-3-0-mm-3D_glrlm_LongRunHighGrayLevelEmphasis1ROI	−0.0695	971.5188	765.1715	0.000*	W	Tumor ROI	Texture	LoG
LoG-sigma-1-0-mm-3D_glcm_Correlation1ROI	0.0694	0.5867	0.0538	0.002	*t*	Tumor ROI	Texture	LoG
LBP-3D-k_glrlm_RunVariance2ROI	0.0655	18.9969	6.2895	0.007*	W	Tumor-to-brain interface ROI	Texture	LBP
original_shape_SurfaceVolumeRatio2ROI	−0.0643	0.3724	0.0438	0.006*	*t*	Tumor-to-brain interface ROI	Shape	Original image
logarithm_glcm_Correlation2ROI	0.0636	0.7629	0.0834	0.000*	*t*	Tumor-to-brain interface ROI	Texture	LoG
LBP-3D-m2_glszm_LargeAreaLowGrayLevelEmphasis2ROI	0.0614	86.305	63.3261	0.001*	W	Tumor-to-brain interface ROI	Texture	LBP
exponential_glcm_Correlation2ROI	0.0581	0.7174	0.1598	0.000*	W	Tumor-to-brain interface ROI	Texture	Exponential
LoG-sigma-2-0-mm-3D_ngtdm_Strength2ROI	−0.0533	5.7185	6.7859	0.009*	W	Tumor-to-brain interface ROI	Texture	LoG
original_firstorder_Minimum1ROI	0.0501	−5.0574	41.7556	0.000*	*t*	Tumor ROI	First-order	Original image
wavelet-HH_glszm_SmallAreaEmphasis1ROI	−0.0495	0.7119	0.0341	0.000*	*t*	Tumor ROI	Texture	Wavelet
exponential_gldm_SmallDependenceLowGrayLevelEmphasis1ROI	−0.0473	0.0231	0.0067	0.000*	W	Tumor ROI	Texture	Exponential
exponential_gldm_LowGrayLevelEmphasis1ROI	−0.0462	0.4158	0.2573	0.000*	W	Tumor ROI	Texture	Exponential
LBP-3D-m2_firstorder_Skewness1ROI	−0.046	−0.595	0.2577	0.000*	W	Tumor ROI	First-order	LBP
wavelet-LL_firstorder_Skewness1ROI	0.0374	−0.3226	0.5985	0.006*	*t*	Tumor ROI	First-order	Wavelet
LBP-3D-k_gldm_LargeDependenceHighGrayLevelEmphasis2ROI	0.0365	61.6663	2.7931	0.000*	*t*	Tumor-to-brain interface ROI	Texture	LBP
original_firstorder_Minimum2ROI	0.0357	−46.631	32.7178	0.000*	*t*	Tumor-to-brain interface ROI	First-order	Original image
LBP-3D-m1_glszm_SmallAreaLowGrayLevelEmphasis1ROI	0.0353	0.1478	0.026	0.008*	*t*	Tumor ROI	Texture	LBP
LBP-3D-k_glcm_Correlation1ROI	0.03	0.2733	0.092	0.004*	*t*	Tumor ROI	Texture	LBP
LBP-3D-m1_glszm_LargeAreaEmphasis2ROI	0.0298	497.883	349.1798	0.000*	W	Tumor-to-brain interface ROI	Texture	LBP
LBP-3D-k_glrlm_RunEntropy1ROI	−0.0266	4.1129	0.2879	0.031*	*t*	Tumor ROI	Texture	LBP
wavelet-LH_gldm_LargeDependenceLowGrayLevelEmphasis1ROI	0.0245	0.016	0.0189	0.000*	W	Tumor ROI	Texture	Wavelet
wavelet-LH_glszm_SizeZoneNonUniformityNormalized2ROI	−0.0196	0.5121	0.0603	0.000*	*t*	Tumor-to-brain interface ROI	Texture	Wavelet
exponential_glrlm_ShortRunLowGrayLevelEmphasis2ROI	−0.0176	0.1056	0.0286	0.005*	W	Tumor-to-brain interface ROI	Texture	Exponential
LBP-3D-k_glszm_LargeAreaHighGrayLevelEmphasis1ROI	−0.016	36,713.5462	24,915.5914	0.021*	*t*	Tumor ROI	Texture	LBP
LoG-sigma-4-0-mm-3D_firstorder_Skewness1ROI	−0.0116	0.0814	0.4851	0.033*	*t*	Tumor ROI	First-order	LoG
logarithm_glrlm_RunLengthNonUniformityNormalized2ROI	−0.0098	0.8039	0.0714	0.000*	*t*	Tumor-to-brain interface ROI	Texture	LoG
logarithm_glcm_Correlation1ROI	0.0094	0.7269	0.1057	0.000*	W	Tumor ROI	Texture	LoG
LBP-3D-k_glszm_ZonePercentage1ROI	0.0064	0.0224	0.0075	0.001*	*t*	Tumor ROI	Texture	LBP
exponential_glcm_Idn2ROI	−0.0031	0.9767	0.011	0.007*	*t*	Tumor-to-brain interface ROI	Texture	Exponential
square_gldm_SmallDependenceLowGrayLevelEmphasis1ROI	−0.0018	0.0124	0.0084	0.000*	W	Tumor ROI	Texture	Square

ROI, region of interest; W, Wilcoxon test; t, t-test. *p < 0.05.

Based on the above features, the LR algorithm was applied to construct the TRM and TbRM by training on each tumoral and tumor-to-brain interface radiomics feature set, respectively, which subsequently converted the output probability scores into radiomics scores (Rscore_1ROI, Rscore_2ROI) by the formula in **Supplement Material 3**.

### 3.3 Multivariate Analysis of LR: Semantic Features and Rscore

Then, all the semantic features and Rscore, including peritumoral edema, tumor location, hyperostosis, T2W signal, and CSF cleft sign, and Rscore_1ROI and Rscore_2ROI, were combined to construct an integrated model, CSRN, by using multivariate analysis of LR. The variance inflation factor (VIF) test was performed. **Table 4** lists all these included features and their statistical data and ranked them according to *P*-values. As a result, the importance order of brain invasion predictors was as follows: peritumoral edema > Rscore_2ROI (tumor-to-brain interface radiomics features) > Rscore_1ROI (tumoral radiomics features) > tumor location > CSF cleft sign > T2-weighted signal > osteogenesis.

**Table 4:** The result of multiple logistic regression.

Features	Coef	*t*/*χ* ^2^	*p*-value
Peritumoral edema	1.3079	42.592	<0.0001
Tumor location	−0.0633	33.021	0.0046**
Hyperostosis	0.8289	7.594	0.0309*
T2-weighted signal	−0.0210	7.075	0.0095**
CSF cleft sign	−1.3991	8.493	0.0063**
Rscore_1ROI	1.5849	−10.338	0.0013**
Rscore_2ROI	4.1189	−7.516	<0.0001

*p < 0.05, **p < 0.01.

### 3.4 The Performance of CSRN, TRM, TbRM, CSM, and TCTbRM

**Figure 6:**
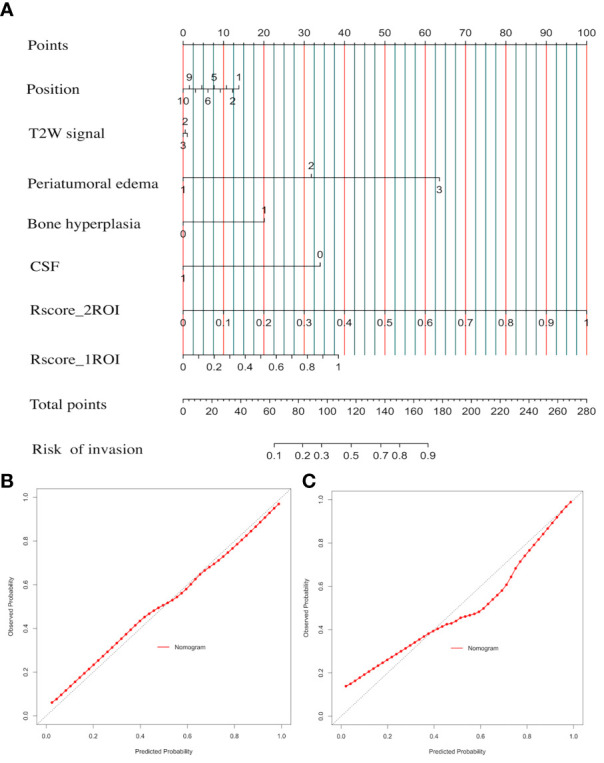
Clinical semantic and radiomics nomogram (CSRN) and its calibration curves. **(A)** Nomogram; **(B)** correction curve of the training set; **(C)** calibration curves of the test set.

CSRN combined seven factors and the LR algorithm to calculate the risk probability of brain invasion for meningioma patients. In **Figure 6A**, the input and output of CSRN had be quantified in the nomogram. According to the value of each patient in each factor, each quantized point (“Point”) would be obtained and the total points were summed (“Total points”), and then the risk of brain invasion was calculated (“Risk of invasion”). The detailed explanation of each factor is shown in **Supplement Material 4**. The higher the total score, the greater the risk of brain invasion of the patient is. We drew nomogram correction curves (**Figures 6B, C** **)** on the training set and the test set, respectively. It can be seen that the prediction curve is close to the reference line (slope = 1), indicating its prediction ability is excellent.

**Figure 7:**
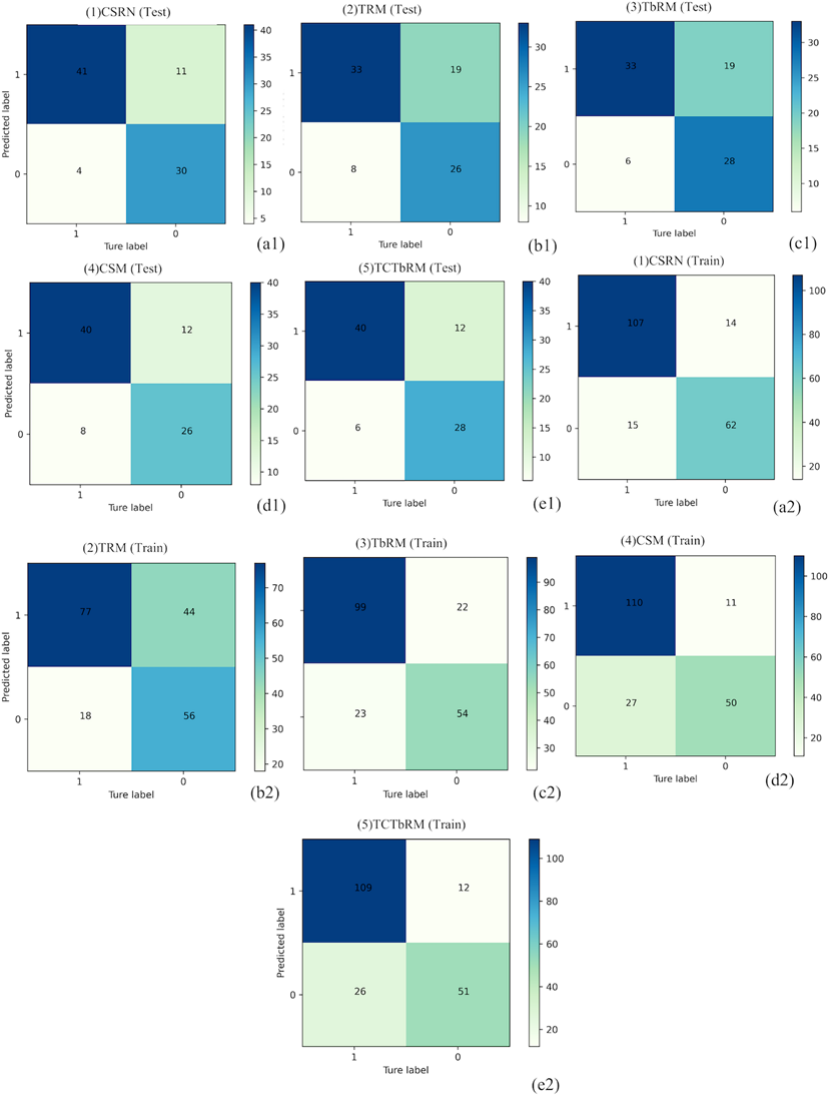
Confusion matrixes of the five models. Test set: CSRN **(A1)**, TRM **(B1)**, TbRM **(C1)**, CSM **(D1)**, and TCTbRM **(E1)**; training set: CSRN **(A2)**, TRM **(B2)**, TbRM **(C2)**, CSM **(D2)**, and TCTbRM **(E2)**. CSRN, clinical semantic and radiomics model/nomogram; TRM, tumoral radiomics model; TbRM, tumor-to-brain interface radiomics model; CSM, clinical semantic model; TCTbRM, tumor combined tumor-to-brain interface radiomics model.

**Figure 8:**
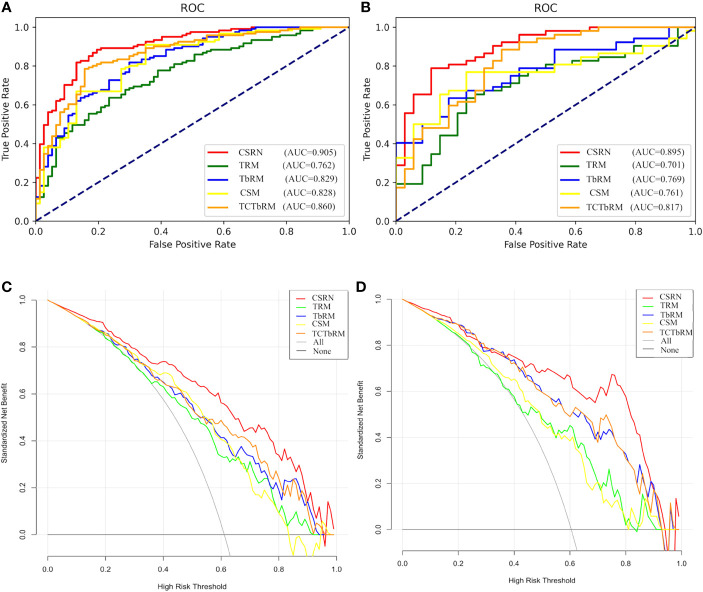
Performance of the five models. **(A)** ROC curve of the training set; **(B)** ROC curve of the test set; **(C)** DCA curve of the training set; **(D)** DCA curve of the test set. ROI, region of interest; DCA, decision curve analysis.

Furthermore, the performances of CSRN and the other four models (TRM, TbRM, CSM, TCTbRM) are shown in **Figure 7**, respectively, by confusion matrix, and it can be seen that the number of false-positive and false-negative samples of CSRN was lower than that of the other models in both training and test sets. The ROC curves and AUCs of the five models in the training set and the test set are, respectively, shown in **Figures 8A, B**, indicating that the AUC of CSRN was the largest.

Youden coefficient was used to find the cutoff point of the ROC curve and to calculate the accuracy, sensitivity, specificity, NPV, and PPV for each model, and all indexes are shown in **Table 5**. In **Supplement Material 5**, we demonstrated the process of using Youden to find the cutoff point on the training set of the CSRN.

**Table 5:** Comparison of the performance of the models.

Model	Training set (*n* = 198)							Test set (*n* = 86)						
	AUC (95% CI)	*p*-value (*vs.* CSRN)	ACC	SEN	SPE	NPV	PPV	AUC (95% CI)	*p*-value *vs.* CSRN	ACC	SEN	SPE	NPV	PPV
CSRN	0.905 (0.863–0.9472)	–	0.854	0.884 (0.813–0.935)	0.81 (0.699–0.887)	0.816 (0.717–0.893)	0.877 (0.800–0.931)	0.895 (0.828–0.962)	–	0.826	0.788 (0.653–0.889)	0.882 (0.725–0.967)	0.732 (0.579–0.914)	0.911 (0.783–0.957)
TRM	0.762 (0.695–0.829)	0.0004**	0.689	0.636 (0.544–0.722)	0.77 (0.656–0.855)	0.573 (0.478–0.707)	0.811 (0.713–0.864)	0.701 (0.588–0.814)	0.004**	0.686	0.635 (0.490–0.764)	0.765 (0.588–0.893)	0.578 (0.430–0.778)	0.805 (0.645–0.885)
TbRM	0.829 (0.771–0.888)	0.039*	0.773	0.818 (0.738–0.882)	0.701 (0.586–0.800)	0.711 (0.605–0.807)	0.812 (0.722–0.878)	0.769 (0.671–0.867)	0.039*	0.709	0.635 (0.490–0.764)	0.84 (0.655–0.932)	0.596 (0.449–0.813)	0.846 (0.691–0.911)
CSM	0.828 (0.769–0.887)	0.037*	0.808	0.909 (0.843–0.954)	0.649 (0.53–0.755)	0.820 (0.710–0.883)	0.803 (0.714–0.894)	0.761 (0.658–0.863)	0.033*	0.767	0.769 (0.632–0.875)	0.765 (0.588–0.893)	0.684 (0.527–0.847)	0.833 (0.687–0.913)
TCTbRM	0.860 (0.807–0.913)	0.072	0.808	0.785 (0.701–0.855)	0.844 (0.744–0.917)	0.714 (0.616–0.836)	0.888 (0.809–0.927)	0.817 (0.723–0.910)	0.046*	0.791	0.885 (0.766–0.956)	0.647 (0.46–0.803)	0.786 (0.610–0.890)	0.793 (0.645–0.917)

ACC, accuracy; SEN, sensitivity; SPE, specificity; NPV, negative predictive value; PPV, positive predictive value.

*Indicates significant difference after the DeLong test. *p< 0.05, **p < 0.01.

The results in **Table 5** show the following: in the training set, CSRN had the highest AUC of 0.905 (95% CI 0.863–0.9472), which was significantly higher than those of the TRM (0.762, 95% CI 0.695–0.829), TbRM (0.829, 95% CI 0.771–0.888), CSM (0.828, 95% CI 0.769–0.887), and TCTbRM (0.860, 95% CI 0.807–0.913), and the AUC of the TCTbRM was better than that of the CSM, while the AUC of the TbRM was close to that of the CSM. Specifically, in the test set, the AUC of CSRN was 0.895 (95% CI 0.828–0.962), which was significantly higher than that of the TRM (0.701, 95% CI 0.588–0.814) and significantly higher than those of the TbRM (0.769, 95% CI 0.67–0.867), CSM (0.761, 95% CI 0.658–0.863), and TCTbRM (0.817, 95% CI 0.723–0.91) (DeLong test, *P* < 0.05).

The accuracy, sensitivity, specificity, NPV, and PPV of CSRN on the test set were 0.826, 0.788, 0.882, 0.732, and 0.911, respectively, among which accuracy, specificity, and NPV were significantly higher than those of all the other models (*Z*-test, *P* < 0.05); the specificity and NPV of TCTbRM were higher than those of CSRN (0.885 *vs.* 0.788, 0.786 *vs.* 0.732) (*Z*-test, *P* < 0.05), while accuracy, specificity, and PPV were lower than those of the CSRN.

In order to explore the auxiliary value of different types of features in making clinical decision, we performed clinical decision analysis (DCA) on different models, and these are shown in **Figures 8C, D** of the training set and test set. The results showed that the clinical net benefit (NB) of CSRN was higher than that of all the other models in the training set. If the prediction probability of 35%–90% was selected as the diagnostic model, the clinical NB of CSRN in the test set is higher than that of all the other models, while when the prediction probability was 20%–35%, the NB of all the models were close.

## 4 Discussion

This study comprehensively extracted high-throughput radiomics features from tumoral and tumor-to-brain interface regions as well as traditional semantic features and also explored the performance in predicting brain invasion in meningioma among different predictive models that were constructed on corresponding radiomics and semantic features. We had two main findings: 1) all the CSM, TRM, and TbRM had significant but similar contributions to predicting brain invasion in meningioma; and 2) an individually available nomogram that was composed of semantic feature set, radiomics feature set of tumor, and tumor-to-brain interface regions was constructed, which had the best prediction of brain invasion in both training and test sets.

In the building of CSM, traditional radiological findings, like peritumoral edema, CSF cleft sign, hyperostosis, T2-weigthed signal, and tumor location, were finally included, suggesting that meningiomas with severe peritumoral edema, loss of CSF cleft sign, obvious hyperostosis, low T2-weighted signal, and anterior fossa base location would have a higher risk of brain invasion. Peritumoral edema is the most important semantic feature in predicting brain invasion of meningioma, which was consistently reported by previous studies (6, 15, 27). As demonstrated in the present study, meningioma with one or several of these findings may be indirectly indicating aggressive biological behavior, e.g., regional infiltration to the brain and bone tissues (the occurrence of peritumoral edema, loss of CSF cleft sign, and hyperostosis) (28), high tumor cell density (low T2-weighted signal), and various tumor microenvironments and histopathological origins in different anatomical locations (29). When estimating this CSM, we observed a moderate performance (AUC = 0.761) in predicting brain invasion in the test dataset. Therefore, it remains active to further improve the performance and facilitate the clinical translation of preoperative MRI.

Radiomics measurements from tumor and related regions have been well established as a promising approach to quantify tumor shapes, intensity distributions, spatial relationships, and texture heterogeneity that are difficult to find on routine imaging and imperceptible to the human eyes (9). Therefore, the current study extracted radiomics features to assist in predicting brain invasion for meningioma by two steps. First, we extracted radiomics features within the tumor region, built TRM, and calculated Rscore to represent its performance in predicting brain invasion individually. The AUCs in training set and test set were 0.762 and 0.701, respectively, which were relatively consistent with a recent study (AUC = 0.682 in the training set and 0.735 in the validation set) by employing enhanced T1-weighted imaging (14). Moreover, several studies hypothesized that the tumor-to-brain interface radiomics features may reflect tumor-associated alterations, e.g., direct tumor involvement and indirect immunoreaction (15, 30). By singly learning tumor-to-brain interface radiomics features, the AUCs of TbRM reached 0.829 and 0.769 in the training set and test set, respectively. However, the prediction performances of TRM, TbRM, and CSM remained moderate, and no intermodel difference was observed among them, which suggested that current protocols were still hard to be potentially translated in clinical practice. Alternatively, it should be worth noting that those three kinds of imaging features were enriched with very different but complementary biological information, i.e., TRM indicated intrinsic tumor property [e.g., spatial heterogeneity of tumor tissue (9)], TbRM specified tumor-related infiltration (15, 30), and CSM provided both tumor and tumor-to-brain interface information in a macroscopic way. Therefore, to advance the study, we improved our protocol by training model from different sets of features that may increase understanding of tumor biology.

Herein, a TCTbRM was constructed and its performance was estimated with AUCs of 0.860 and 0.817 in the training set and test set. This radiomics model comprehensively explained tumor behavior in a voxel-to-voxel way. Although the model performance was not significantly better than that mentioned above, a trend of increased prediction efficacy was indicated with TCTbRM > TbRM ≈ CSM > TRM in the test set. However, to the best of our knowledge, such radiomics model was not included following information, but CSM provided the following: 1) the relationship with neighboring tissues (e.g., bone) cannot be considered, 2) the distal and severe edema related to tumor was ignored since only 5 mm from the tumor margin was estimated, and 3) the tumor tissue origin may be different from intracranial sites. Therefore, a prediction model (CSRN) that combined all three kinds of tumor features was constructed, and a significant improvement in performance was observed (AUCs were 0.905 in the training set and 0.895 in the test dataset). A nomogram was then built that quantified the risk point of each semantic feature and Rscore from tumoral and tumor-to-brain interface radiomics. Furthermore, DCA demonstrated that, with the assistance of CSRN, radiologists would obtain higher clinical benefits in clinical decision-making.

This study had several limitations. First, the pathological diagnosis of brain invasion may be subject to sampling error, especially when diagnosing meningioma without brain invasion. In our study, all patients with brain invasion were confirmed by pathological evidence; however, the diagnosis of negative cases may be to some extent associated with insufficient tissue blocks during operation. Therefore, future radiologic–pathologic association analysis would be helpful to confirm the present findings. Second, even though this study included all meningioma patients with brain invasion from 2011 to 2020 with pathological confirmation, the sample size was relatively small and only single-center data were available. Therefore, it is promising to make CSRN go through multicenter dataset with a larger sample size in the future. Third, the enlargement of features in the model construction may cause overfitting; here, we reduce the overfitting risk by randomly splitting the dataset into training set and independent test set. In the future, more external validations are warranted. Fourth, although we performed image preprocessing to minimize the variability, including *Z*-score normalization and B-spline interpolation sampling method, the MRI data used in the present study were acquired using different scanners, which may bring some biases. In reverse, as there was no correction by scanner type, this illustrates the translational potential of our results and it is a strong argument in favor of a multicentric application of radiomics.

In conclusion, this study firstly disclosed that traditional semantic findings had comparable performance in predicting brain invasion of meningioma with radiomics information. By taking advantage of semantic features and radiomics features from tumoral and tumor-to-brain interface regions, an integrated nomogram model was constructed that had excellent efficacy in predicting brain invasion, which currently was available for further clinical validation.

## Data Availability Statement

The raw data supporting the conclusions of this article will be made available by the authors, without undue reservation.

## Ethics Statement

The studies involving human participants were reviewed and approved by the Second Affiliated Hospital of Zhejiang University School of Medicine. Written informed consent for participation was not required for this study in accordance with the national legislation and the institutional requirements.

## Author Contributions

XX and XG conceived the project. XX, XG, and NL designed the experiments. XX, XG, NL, MH, XW, JW, SY, HW, FD, and MZ acquired the MRI data. XX, XG, NL, YM, CH, KH, MH, XW, JW, SY, HW, FD, FS, YL, and YY performed the experiments. XX, XG, NL, and YM wrote the manuscript. All authors contributed to the article and approved the submitted version.

## Funding

This study was supported by the National Key Research and Development Program of China (Grant No. 2017YFC0113400), Natural Science Foundation of Zhejiang Province (Grant No. LQ21H180008) and the China Postdoctoral Science Foundation (Grant No. 2021T140599 and 2019M662082).

## Conflict of Interest

The authors YM, CH, KH, FS, YL, and YY were employed by Beijing Deepwise & League of PHD Technology Co., Ltd.

The remaining authors declare that the research was conducted in the absence of any commercial or financial relationships that could be construed as a potential conflict of interest.

## Publisher’s Note

All claims expressed in this article are solely those of the authors and do not necessarily represent those of their affiliated organizations, or those of the publisher, the editors and the reviewers. Any product that may be evaluated in this article, or claim that may be made by its manufacturer, is not guaranteed or endorsed by the publisher.
