# Supplementary material for: A Clinical Semantic and Radiomics Nomogram for Predicting Brain Invasion in WHO Grade II Meningioma Based on Tumor and Tumor-to-Brain Interface Features
Source: Front Oncol. 2021 Oct 22;11:752158. doi: 10.3389/fonc.2021.752158 (PMC8570084; doi:10.3389/fonc.2021.752158)
Supplement: Supplementary file 1 [file DataSheet_1.docx]

Features in this study are named as: Image processing method _ feature category_name _ROI , such as the”log-sigma-3-0-mm-3D_glrlm_ShortRunLowGrayLevelEmphasis2ROI” , said after the log transformation from tumoral ROI (sigma =3.0) 3D texture features (GLRLM category) Short Run Low Gray Level Cast.

**Explanation of radiomics features**

## first-order feature

|  | **Name** | **Explanation** |
| --- | --- | --- |
| 1 | Energy | Energy is a measure of the magnitude of voxel values in an image. A larger values implies a greater sum of the squares of these values. |
| 2 | Total Energy | Total Energy is the value of Energy feature scaled by the volume of the voxel in cubic mm. |
| 3 | Entropy | Entropy specifies the uncertainty/randomness in the image values. |
| 4 | Minimum | The minimum value of a set of voxels included in the ROI |
| 5 | 10th percentile | The 10^th^ percentile of a set of voxels included in the ROI |
| 6 | 10th percentile | The 90^th^ percentile of a set of voxels included in the ROI |
| 7 | Maximum | The maximum gray level intensity within the ROI. |
| 8 | Mean | The median gray level intensity within the ROI |
| 9 | **Interquartile Range** | P25 and P75 are the 25^th^ and 75^th^ percentile of the image array, respectively |
| 10 | **range** | The range of gray values in the ROI. |
| 11 | MAD | Mean Absolute Deviation is the mean distance of all intensity values from the Mean Value of the image array |
| 12 | rMAD | Robust Mean Absolute Deviation is the mean distance of all intensity values from the Mean Value calculated on the subset of image array with gray levels in between, or equal to the 10^th^ and 90^th^ percentile. |
| 13 | RMS | RMS is the square-root of the mean of all the squared intensity values. It is another measure of the magnitude of the image values. |
| 14 | Standard Deviation | The amount of variation or dispersion from the Mean Value |
| 15 | Kurtosis | A measure of the ‘peakedness’ of the distribution of values in the image ROI. A higher kurtosis implies that the mass of the distribution is concentrated towards the tail(s) rather than towards the mean. A lower kurtosis implies the reverse: that the mass of the distribution is concentrated towards a spike near the Mean value. |
| 16 | Variance | The mean of the squared distances of each intensity value from the Mean value. This is a measure of the spread of the distribution about the mean |
| 17 | Uniformity | A measure of the sum of the squares of each intensity value. This is a measure of the homogeneity of the image array, where a greater uniformity implies a greater homogeneity or a smaller range of discrete intensity values |

## (3D) Shape Features

|  | **Name** | **Explanation** |
| --- | --- | --- |
| 1 | Mesh Volume | The volume of the ROI V is calculated from the triangle mesh of the ROI. |
| 2 | Voxel Volume | This is a less precise approximation of the volume and is not used in subsequent features. This feature does not make use of the mesh and is not used in calculation of other shape features. |
| 3 | **Surface Area** | The total surface area is obtained by taking the sum of all calculated sub-areas |
| 4 | **Surface Area to Volume ratio** | A lower value indicates a more compact (sphere-like) shape. |
| 5 | Sphericity | A measure of the roundness of the shape of the tumor region relative to a sphere. The value range is 0<sphericity≤1, where a value of 1 indicates a perfect sphere |
| 6 | Compactness 1 | Similar to Sphericity, Compactness 1 is a measure of how compact the shape of the tumor is relative to a sphere (most compact). The value range is 0<compactness ≤1/16π, where a value of 1/16π indicates a perfect sphere. |
| 7 | Compactness 2 | Similar to Sphericity and Compactness 1, Compactness 2 is a measure of how compact the shape of the tumor is relative to a sphere (most compact). It is a dimensionless measure, independent of scale and orientation. |
| 8 | Spherical Disproportion | Spherical Disproportion is the ratio of the surface area of the tumor region to the surface area of a sphere with the same volume as the tumor region, and by definition, the inverse of Sphericity. Therefore, the value range is spherical disproportion≥1, with a value of 1 indicating a perfect sphere. |
| 9 | Maximum 3D diameter | Maximum 3D diameter is defined as the largest size of the tumor. |
| 10 | **Maximum 2D diameter (Slice)** | As the largest size of tumor surface mesh vertices in the row-column (generally the axial) plane. |
| 11 | **Maximum 2D diameter (Column)** | Maximum 2D diameter (Column) is defined as the largest pairwise Euclidean distance between tumor surface mesh vertices in the row-slice (usually the coronal) plane. |
| 12 | Maximum 2D diameter (Row) | Maximum 2D diameter (Row) is defined as the largest pairwise Euclidean distance between tumor surface mesh vertices in the column-slice (usually the sagittal) plane. |
| 13 | Major Axis Length | This feature yield the largest axis length of the ROI-enclosing ellipsoid and is calculated using the largest principal component λmajor |
| 14 | Minor Axis Length | This feature yield the second-largest axis length of the ROI-enclosing ellipsoid |
| 15 | Least Axis Length | This feature yield the smallest axis length of the ROI-enclosing ellipsoid. In case of a 2D segmentation, this value will be 0. |
| 16 | Elongation | Elongation shows the relationship between the two largest principal components in the ROI shape. |
| 17 | Flatness | Flatness shows the relationship between the largest and smallest principal components in the ROI shape. |

## （2D）Shape Features

|  | **Name** | **Explanation** |
| --- | --- | --- |
| 1 | **Mesh Surface** | The surface area of all the tumors |
| 2 | Pixel Surface | This is a less precise approximation of the surface area. |
| 3 | **Perimeter** | The circumference of the tumor |
| 4 | Perimeter to Surface ratio | A lower value indicates a more compact (circle-like) shape. This feature is not dimensionless, and is therefore (partly) dependent on the surface area of the ROI. |
| 5 | Sphericity | A measure of the roundness of the shape of the tumor region relative to a sphere. The value range is 0<sphericity≤1, where a value of 1 indicates a perfect sphere |
| 6 | Spherical Disproportion | Spherical Disproportion is the ratio of the surface area of the tumor region to the surface area of a sphere with the same volume as the tumor region, and by definition, the inverse of Sphericity. Therefore, the value range is spherical disproportion≥1, with a value of 1 indicating a perfect sphere. |
| 7 | **Maximum 2D diameter (Slice)** | As the largest size of tumor surface mesh vertices in the row-column (generally the axial) plane. |
| 8 | Major Axis Length | This feature yield the largest axis length of the ROI-enclosing ellipsoid and is calculated using the largest principal component λmajor |
| 9 | Minor Axis Length | This feature yield the second-largest axis length of the ROI-enclosing ellipsoid |
| 10 | Elongation | Elongation shows the relationship between the two largest principal components in the ROI shape. |

## GLCM

|  | **Name** | **Explanation** |
| --- | --- | --- |
| 1 | **Autocorrelation** | A measure of the magnitude of the fineness and coarseness of texture. |
| 2 | **Joint Average** | Returns the mean gray level intensity |
| 3 | **Cluster Prominence** | A measure of the skewness and asymmetry of the GLCM. A higher values implies more asymmetry about the mean while a lower value indicates a peak near the mean value and less variation about the mean. |
| 4 | **Cluster Shade** | A measure of the skewness and uniformity of the GLCM. A higher cluster shade implies greater asymmetry about the mean. |
| 5 | **Cluster Tendency** | **A measure of groupings of voxels with similar gray-level values** |
| 6 | **Contrast** | A measure of the local intensity variation A larger value correlates with a greater disparity in intensity values among neighboring voxels. |
| 7 | **Correlation** | A value between 0 (uncorrelated) and 1 (perfectly correlated) showing the linear dependency of gray level values to their respective voxels |
| 8 | Difference Average | Difference Average measures the relationship between occurrences of pairs with similar intensity values and occurrences of pairs with differing intensity values. |
| 9 | **Difference Entropy** | A measure of the randomness/variability in neighborhood intensity value differences |
| 10 | **Difference Variance** | Difference Variance is a measure of heterogeneity that places higher weights on differing intensity level pairs that deviate more from the mean. |
| 11 | **Joint Energy** | A measure of homogeneous patterns in the image. A greater Energy implies that there are more instances of intensity value pairs in the image that neighbor each other at higher frequencies. |
| 12 | **Joint Entropy** | **A measure of the randomness/variability in neighborhood intensity values.** |
| 13 | **Informational Measure of Correlation (IMC) 1** | Quantify the complexity of textures |
| 14 | **Informational Measure of Correlation (IMC) 2** |  |
| 15 | **Inverse Difference Moment (IDM)** | A measure of the local homogeneity of an image |
| 16 | **Maximal Correlation Coefficient (MCC)** | A measure of complexity of the texture |
| 17 | **Inverse Difference Moment Normalized (IDMN)** | A measure of the local homogeneity of an image. |
| 18 | **Inverse Difference (ID)** | Another measure of the local homogeneity of an image. With more uniform gray levels, the denominator will remain low, resulting in a higher overall value. |
| 19 | **Inverse Difference Normalized (IDN)** | Another measure of the local homogeneity of an image. |
| 20 | **Inverse Variance** | **Inverse Variance** |
| 21 | **Maximum Probability** | Occurrences of the most predominant pair of neighboring intensity values. |
| 22 | **Sum Average** | Measures the relationship between occurrences of pairs with lower intensity values and occurrences of pairs with higher intensity values. |
| 23 | **Sum Entropy** | A sum of neighborhood intensity value differences. |
| 24 | **Sum of Squares** | A measure in the distribution of neighboring intensity level pairs about the mean intensity level |

## 5、GLSZM

The gray level region of the quantized image is defined as the number of adjacent voxels with the same gray intensity

|  | **Name** | **Explanation** |
| --- | --- | --- |
| 1 | Small Area Emphasis (SAE) | SAE is a measure of the distribution of small size zones, with a greater value indicative of smaller size zones and more fine textures. |
| 2 | Large Area Emphasis (LAE) | LAE is a measure of the distribution of large area size zones, with a greater value indicative of larger size zones and more coarse textures. |
| 3 | Gray Level Non-Uniformity (GLN**)** | GLN measures the variability of gray-level intensity values in the image, with a lower value indicating more homogeneity in intensity values. |
| 4 | Gray Level Non-Uniformity Normalized (GLNN) | GLNN measures the variability of gray-level intensity values in the image, with a lower value indicating a greater similarity in intensity values. This is the normalized version of the GLN formula. |
| 5 | Size-Zone Non-Uniformity (SZN) | SZN measures the variability of size zone volumes in the image, with a lower value indicating more homogeneity in size zone volumes. |
| 6 | Size-Zone Non-Uniformity Normalized (SZNN) | SZNN measures the variability of size zone volumes throughout the image, with a lower value indicating more homogeneity among zone size volumes in the image. This is the normalized version of the SZN formula. |
| 7 | Zone Percentage (ZP) | ZP measures the coarseness of the texture by taking the ratio of number of zones and number of voxels in the ROI. |
| 8 | Gray Level Variance (GLV) | GLV measures the variance in gray level intensities for the zones. |
| 9 | Zone Variance (ZV) | ZV measures the variance in zone size volumes for the zones. |
| 10 | Zone Entropy (ZE) | ZE measures the uncertainty/randomness in the distribution of zone sizes and gray levels. A higher value indicates more heterogeneneity in the texture patterns. |
| 11 | Low Gray Level Zone Emphasis (LGLZE) | LGLZE measures the distribution of lower gray-level size zones, with a higher value indicating a greater proportion of lower gray-level values and size zones in the image. |
| 12 | High Gray Level Zone Emphasis (HGLZE) | HGLZE measures the distribution of the higher gray-level values, with a higher value indicating a greater proportion of higher gray-level values and size zones in the image. |
| 13 | Small Area Low Gray Level Emphasis (SALGLE) | SALGLE measures the proportion in the image of the joint distribution of smaller size zones with lower gray-level values. |
| 14 | Small Area High Gray Level Emphasis (SAHGLE) | SAHGLE measures the proportion in the image of the joint distribution of smaller size zones with higher gray-level values |
| 15 | Large Area Low Gray Level Emphasis (LALGLE) | LALGLE measures the proportion in the image of the joint distribution of larger size zones with lower gray-level values |
| 16 | Large Area High Gray Level Emphasis (LAHGLE) | LAHGLE measures the proportion in the image of the joint distribution of larger size zones with higher gray-level values |

###

## 6、GLRLM

Used to describe the distribution of pixel values

|  | **Name** | **Explanation** |
| --- | --- | --- |
| 1 | **Short Run Emphasis** | SRE is a measure of the distribution of short run lengths, with a greater value indicative of shorter run lengths and more fine textural textures. |
| 2 | **Long Run Emphasis** | LRE is a measure of the distribution of long run lengths, with a greater value indicative of longer run lengths and more coarse structural textures. |
| 3 | **Gray Level Non-Uniformity (GLN)** | GLN measures the similarity of gray-level intensity values in the image, where a lower GLN value correlates with a greater similarity in intensity values. |
| 4 | **Gray Level Non-Uniformity Normalized (GLNN)** | GLNN measures the similarity of gray-level intensity values in the image, where a lower GLNN value correlates with a greater similarity in intensity values. This is the normalized version of the GLN formula. |
| 5 | **Run Length Non-Uniformity (RLN)** | similarity of run lengths throughout the image, with a lower value indicating more homogeneity among run lengths in the image. |
| 6 | **Run Length Non-Uniformity Normalized (RLNN)** | RLNN measures the similarity of run lengths throughout the image, with a lower value indicating more homogeneity among run lengths in the image. This is the normalized version of the RLN formula. |
| 7 | **Run Percentage (RP)** | RP measures the coarseness of the texture by taking the ratio of number of runs and number of voxels in the ROI. |
| 8 | **Gray Level Variance (GLV)** | GLV measures the variance in gray level intensity for the runs. |
| 9 | **Run Variance (RV)** | RV is a measure of the variance in runs for the run lengths. |
| 10 | **Run Entropy (RE)** | RE measures the uncertainty/randomness in the distribution of run lengths and gray levels. A higher value indicates more heterogeneity in the texture patterns. |
| 11 | **Low Gray Level Run Emphasis (LGLRE)** | LGLRE measures the distribution of low gray-level values, with a higher value indicating a greater concentration of low gray-level values in the image. |
| 12 | **High Gray Level Run Emphasis (HGLRE)** | HGLRE measures the distribution of the higher gray-level values, with a higher value indicating a greater concentration of high gray-level values in the image. |
| 13 | **Short Run Low Gray Level Emphasis (SRLGLE)** | SRLGLE measures the joint distribution of shorter run lengths with lower gray-level values. |
| 14 | **Short Run High Gray Level Emphasis (SRHGLE)** | SRHGLE measures the joint distribution of shorter run lengths with higher gray-level values |
| 15 | **Long Run Low Gray Level Emphasis (LRLGLE)** | LRLGLRE measures the joint distribution of long run lengths with lower gray-level values. |
| 16 | **Long Run High Gray Level Emphasis (LRHGLE)** | LRHGLRE measures the joint distribution of long run lengths with higher gray-level values. |

## 7、GLDM

Represents the grayscale correlation in the image

|  | **Name** | **Explanation** |
| --- | --- | --- |
| 1 | **Small Dependence Emphasis (SDE)** | A measure of the distribution of small dependencies, with a greater value indicative of smaller dependence and less homogeneous textures |
| 2 | **Large Dependence Emphasis (LDE)** | A measure of the distribution of large dependencies, with a greater value indicative of larger dependence and more homogeneous textures. |
| 3 | **Gray Level Non-Uniformity (GLN)** | Measures the similarity of gray-level intensity values in the image, where a lower GLN value correlates with a greater similarity in intensity values |
| 4 | **Dependence Non-Uniformity (DN)** | Measures the similarity of dependence throughout the image, with a lower value indicating more homogeneity among dependencies in the image |
| 5 | **Dependence Non-Uniformity Normalized (DNN)** | Measures the similarity of dependence throughout the image, with a lower value indicating more homogeneity among dependencies in the image. This is the normalized version of the DLN formula |
| 6 | **Gray Level Variance (GLV)** | Measures the variance in grey level in the image. |
| 7 | **Dependence Variance (DV)** | Measures the variance in dependence size in the image. |
| 8 | **Low Gray Level Emphasis (LGLE)** | Measures the distribution of low gray-level values, with a higher value indicating a greater concentration of low gray-level values in the image. |
| 9 | **High Gray Level Emphasis (HGLE)** | Measures the distribution of the higher gray-level values, with a higher value indicating a greater concentration of high gray-level values in the image. |
| 10 | **Small Dependence Low Gray Level Emphasis (SDLGLE)** | Measures the joint distribution of small dependence with lower gray-level values. |
| 11 | **Small Dependence High Gray Level Emphasis (SDHGLE)** | Measures the joint distribution of small dependence with higher gray-level values |
| 12 | **Large Dependence Low Gray Level Emphasis (LDLGLE)** | Measures the joint distribution of large dependence with lower gray-level values |
| 13 | **Large Dependence High Gray Level Emphasis (LDHGLE)** | Measures the joint distribution of large dependence with higher gray-level values |
