# Supplementary material for: A Clinical Semantic and Radiomics Nomogram for Predicting Brain Invasion in WHO Grade II Meningioma Based on Tumor and Tumor-to-Brain Interface Features
Source: Front Oncol. 2021 Oct 22;11:752158. doi: 10.3389/fonc.2021.752158 (PMC8570084; doi:10.3389/fonc.2021.752158)
Supplement: Supplementary file 2 [file DataSheet_2.docx]

**The constituent factors of the five different models**

1. **Tumoral Radiomics Model (TRM):**

Twenty significant tumoral radiomics features were included (**Table 4.1**).

**Table 4.1**

| **Features** | **Weight** | **Mean** | **SD** | **P value** |
| --- | --- | --- | --- | --- |
| logarithm_ngtdm_Busyness1ROI | 0.0942 | 0.0975 | 0.1218 | 0.000* |
| logarithm_ngtdm_Strength1ROI | -0.0882 | 26.2279 | 32.0014 | 0.000* |
| log-sigma-1-0-mm-3D_glcm_InverseVariance1ROI | 0.0828 | 0.4085 | 0.0516 | 0.000* |
| log-sigma-3-0-mm-3D_glrlm_LongRunHighGrayLevelEmphasis1ROI | -0.0695 | 971.5188 | 765.1715 | 0.000* |
| log-sigma-1-0-mm-3D_glcm_Correlation1ROI | 0.0694 | 0.5867 | 0.0538 | 0.002 |
| original_firstorder_Minimum1ROI | 0.0501 | -5.0574 | 41.7556 | 0.000* |
| wavelet-HH_glszm_SmallAreaEmphasis1ROI | -0.0495 | 0.7119 | 0.0341 | 0.000* |
| exponential_gldm_SmallDependenceLowGrayLevelEmphasis1ROI | -0.0473 | 0.0231 | 0.0067 | 0.000* |
| exponential_gldm_LowGrayLevelEmphasis1ROI | -0.0462 | 0.4158 | 0.2573 | 0.000* |
| lbp-3D-m2_firstorder_Skewness1ROI | -0.046 | -0.595 | 0.2577 | 0.000* |
| wavelet-LL_firstorder_Skewness1ROI | 0.0374 | -0.3226 | 0.5985 | 0.006* |
| lbp-3D-m1_glszm_SmallAreaLowGrayLevelEmphasis1ROI | 0.0353 | 0.1478 | 0.026 | 0.008* |
| lbp-3D-k_glcm_Correlation1ROI | 0.03 | 0.2733 | 0.092 | 0.004* |
| lbp-3D-k_glrlm_RunEntropy1ROI | -0.0266 | 4.1129 | 0.2879 | 0.031* |
| wavelet-LH_gldm_LargeDependenceLowGrayLevelEmphasis1ROI | 0.0245 | 0.016 | 0.0189 | 0.000* |
| lbp-3D-k_glszm_LargeAreaHighGrayLevelEmphasis1ROI | -0.016 | 36713.5462 | 24915.5914 | 0.021* |
| log-sigma-4-0-mm-3D_firstorder_Skewness1ROI | -0.0116 | 0.0814 | 0.4851 | 0.033* |
| logarithm_glcm_Correlation1ROI | 0.0094 | 0.7269 | 0.1057 | 0.000* |
| lbp-3D-k_glszm_ZonePercentage1ROI | 0.0064 | 0.0224 | 0.0075 | 0.001* |
| square_gldm_SmallDependenceLowGrayLevelEmphasis1ROI | -0.0018 | 0.0124 | 0.0084 | 0.000* |

1. **Tumor-to-brain Interface Radiomics Model (TbRM):**

Twenty significant tumor-to-brain interface radiomics features were included (**Table 4.2**).

**Table 4.2**

| **Features** | **Weight** | **Mean** | **SD** | **P value** |
| --- | --- | --- | --- | --- |
| log-sigma-3-0-mm-3D_glrlm_ShortRunLowGrayLevelEmphasis2ROI | 0.1468 | 0.0069 | 0.0058 | 0.001* |
| lbp-3D-m2_ngtdm_Complexity2ROI | -0.1455 | 3.8204 | 1.2285 | 0.004* |
| exponential_gldm_SmallDependenceLowGrayLevelEmphasis2ROI | -0.1226 | 0.0199 | 0.0024 | 0.000* |
| square_gldm_SmallDependenceLowGrayLevelEmphasis2ROI | -0.1015 | 0.0228 | 0.0057 | 0.000* |
| log-sigma-4-0-mm-3D_firstorder_Kurtosis2ROI | -0.0935 | 2.9566 | 0.8304 | 0.000* |
| log-sigma-3-0-mm-3D_glcm_Correlation2ROI | 0.0923 | 0.9341 | 0.0101 | 0.000* |
| log-sigma-3-0-mm-3D_glcm_Idmn2ROI | -0.0921 | 0.996 | 0.0016 | 0.007* |
| lbp-3D-k_glrlm_RunVariance2ROI | 0.0655 | 18.9969 | 6.2895 | 0.007* |
| original_shape_SurfaceVolumeRatio2ROI | -0.0643 | 0.3724 | 0.0438 | 0.006* |
| logarithm_glcm_Correlation2ROI | 0.0636 | 0.7629 | 0.0834 | 0.000* |
| lbp-3D-m2_glszm_LargeAreaLowGrayLevelEmphasis2ROI | 0.0614 | 86.305 | 63.3261 | 0.001* |
| exponential_glcm_Correlation2ROI | 0.0581 | 0.7174 | 0.1598 | 0.000* |
| log-sigma-2-0-mm-3D_ngtdm_Strength2ROI | -0.0533 | 5.7185 | 6.7859 | 0.009* |
| lbp-3D-k_gldm_LargeDependenceHighGrayLevelEmphasis2ROI | 0.0365 | 61.6663 | 2.7931 | 0.000* |
| original_firstorder_Minimum2ROI | 0.0357 | -46.631 | 32.7178 | 0.000* |
| lbp-3D-m1_glszm_LargeAreaEmphasis2ROI | 0.0298 | 497.883 | 349.1798 | 0.000* |
| wavelet-LH_glszm_SizeZoneNonUniformityNormalized2ROI | -0.0196 | 0.5121 | 0.0603 | 0.000* |
| exponential_glrlm_ShortRunLowGrayLevelEmphasis2ROI | -0.0176 | 0.1056 | 0.0286 | 0.005* |
| logarithm_glrlm_RunLengthNonUniformityNormalized2ROI | -0.0098 | 0.8039 | 0.0714 | 0.000* |
| exponential_glcm_Idn2ROI | -0.0031 | 0.9767 | 0.011 | 0.007* |

1. **Clinical Semantic Model (CSM):**

Five significant clinical and semantic features were included (**Table 4.3**).

**Table 4.3**

| **Features** |
| --- |
| Peritumoral edema |
| Tumor location |
| Hyperostosis |
| T2-weighted signal |
| CSF cleft sign |

1. **Tumor Combined Tumor-to-brain Interface Radiomics Model (TCTbRM):**

Twenty significant clinical and semantic features were included. See Table 4.1 and Table 4.2.

**5)** **Clinical Semantic of tumor combined with peritumor Radiomics Nomogram (CSRN):**

Five significant clinical semantic features ,Rscore_1ROI and Rscore_2ROI were included（**Table 4.4**）.

**Table 4.4**

| **Features** |
| --- |
| Peritumoral edema |
| Tumor location |
| Hyperostosis |
| T2-weighted signal |
| CSF cleft sign |
| Rscore_1ROI |
| Rscore_2ROI |
