# Supplementary material for: A Clinical Semantic and Radiomics Nomogram for Predicting Brain Invasion in WHO Grade II Meningioma Based on Tumor and Tumor-to-Brain Interface Features
Source: Front Oncol. 2021 Oct 22;11:752158. doi: 10.3389/fonc.2021.752158 (PMC8570084; doi:10.3389/fonc.2021.752158)
Supplement: Supplementary file 3 [file DataSheet_3.docx]

$$Rad\_score=\frac{1}{1+exp(-\sum\beta_{i}*f_{i})}$$

*“Rscore_1ROI=1/{1+exp{-{0.0942*logarithm_ngtdm_Busyness1ROI +(-0.0882)*logarithm_ngtdm_Strength1ROI+0.0828*log-sigma-1-0-mm-3D_glcm_InverseVariance1ROI+(-0.0695)*log-sigma-3-0-mm-3D_glrlm_LongRunHighGrayLevelEmphasis1ROI+0.0694*log-sigma-1-0-mm-3D_glcm_Correlation1ROI+0.0501*original_firstorder_Minimum1ROI+(-0.0495)*wavelet-HH_glszm_SmallAreaEmphasis1ROI+(-0.0473)*exponential_gldm_SmallDependenceLowGrayLevelEmphasis1ROI+(-0.0462)*exponential_gldm_LowGrayLevelEmphasis1ROI+(-0.046)*lbp-3D-m2_firstorder_Skewness1ROI+0.0374*wavelet-LL_firstorder_Skewness1ROI+0.0353*lbp-3D-m1_glszm_SmallAreaLowGrayLevelEmphasis1ROI+0.03*lbp-3D-k_glcm_Correlation1ROI+(-0.0266)*lbp-3D-k_glrlm_RunEntropy1ROI+0.0245*wavelet-LH_gldm_LargeDependenceLowGrayLevelEmphasis1ROI+(-0.016)*lbp-3D-k_glszm_LargeAreaHighGrayLevelEmphasis1ROI+(-0.0116)*log-sigma-4-0-mm-3D_firstorder_Skewness1ROI+0.0094*logarithm_glcm_Correlation1ROI+0.0064*lbp-3D-k_glszm_ZonePercentage1ROI+(-0.0018)*square_gldm_SmallDependenceLowGrayLevelEmphasis1ROI}}”*

*“Rscore_2ROI=1/{1+exp{-{0.1468*log-sigma-3-0-mm-3D_glrlm_ShortRunLowGrayLevelEmphasis2ROI+（-0.1455）*

**lbp-3D-m2_ngtdm_Complexity2ROI+(-0.1226)*exponential_gldm_SmallDependenceLowGrayLevelEmphasis2ROI+(-0.1015)*square_gldm_SmallDependenceLowGrayLevelEmphasis2ROI+(-0.0935)*log-sigma-4-0-mm-3D_firstorder_Kurtosis2ROI+0.0923*log-sigma-3-0-mm-3D_glcm_Correlation2ROI+(-0.0921)*log-sigma-3-0-mm-3D_glcm_Idmn2ROI+0.0655*lbp-3D-k_glrlm_RunVariance2ROI+(-0.0643)*original_shape_SurfaceVolumeRatio2ROI+0.0636*logarithm_glcm_Correlation2ROI+0.0614*lbp-3D-m2_glszm_LargeAreaLowGrayLevelEmphasis2ROI+0.0581*exponential_glcm_Correlation2ROI+(-0.0533)*log-sigma-2-0-mm-3D_ngtdm_Strength2ROI+0.0298*lbp-3D-m1_glszm_LargeAreaEmphasis2ROI+(-0.0196)*wavelet-LH_glszm_SizeZoneNonUniformityNormalized2ROI+(-0.0176)*exponential_glrlm_ShortRunLowGrayLevelEmphasis2ROI+0.0365*lbp-3D-k_gldm_LargeDependenceHighGrayLevelEmphasis2ROI+0.0357*original_firstorder_Minimum2ROI+(-0.0098)*logarithm_glrlm_RunLengthNonUniformityNormalized2ROI+(-0.0031)*exponential_glcm_Idn2ROI}}”*
