# Supplementary material for: A Clinical Semantic and Radiomics Nomogram for Predicting Brain Invasion in WHO Grade II Meningioma Based on Tumor and Tumor-to-Brain Interface Features
Source: Front Oncol. 2021 Oct 22;11:752158. doi: 10.3389/fonc.2021.752158 (PMC8570084; doi:10.3389/fonc.2021.752158)
Supplement: Supplementary file 4 [file DataSheet_4.doc]

**A detailed explanation of each factor in the CSRN model**

| **Factor** | **Interpret** | **Type** |
| --- | --- | --- |
| **Peritumoral edema** | 1 None  2 Mild  3 Marked | Rank variable |
| **Tumor location** | 1 Anterior cranial fossa  2 Middle cranial fossa  3 Posterior cranial fossa  4 Sphenoid crest  5 Sphenoid crest  6 Lateral convexity  7 Midline convexity  8 Tentorium cerebelli  9 Ventricle  10 Other | [C](../../../../C:/Program%20Files%20(x86)/Youdao/Dict/8.9.6.0/resultui/html/index.html#/javascript:;)ategorical [variable](../../../../C:/Program%20Files%20(x86)/Youdao/Dict/8.9.6.0/resultui/html/index.html#/javascript:;) |
| **Hyperostosis** | 0 No  1 Yes | [C](../../../../C:/Program%20Files%20(x86)/Youdao/Dict/8.9.6.0/resultui/html/index.html#/javascript:;)ategorical [variable](../../../../C:/Program%20Files%20(x86)/Youdao/Dict/8.9.6.0/resultui/html/index.html#/javascript:;) |
| **T2** | 1 Hyperintense  2 Isointense  3 Isointense | Rank variable |
| **CSF cleft sign** | 0 No  1 Yes | [C](../../../../C:/Program%20Files%20(x86)/Youdao/Dict/8.9.6.0/resultui/html/index.html#/javascript:;)ategorical [variable](../../../../C:/Program%20Files%20(x86)/Youdao/Dict/8.9.6.0/resultui/html/index.html#/javascript:;) |
| **Rscore_1ROI** | The omics score was calculated based on 20 significant tumor radiomics features | Continuous variable |
| **Rscore_2ROI** | The omics score was calculated based on 20 significant peritumoral radiomics features | Continuous variable |
