# Supplementary figures and images for: A Clinical Semantic and Radiomics Nomogram for Predicting Brain Invasion in WHO Grade II Meningioma Based on Tumor and Tumor-to-Brain Interface Features
Source: Front Oncol. 2021 Oct 22;11:752158. doi: 10.3389/fonc.2021.752158 (PMC8570084; doi:10.3389/fonc.2021.752158)

**The process of calculating cutpoints according to Youden coefficients.**


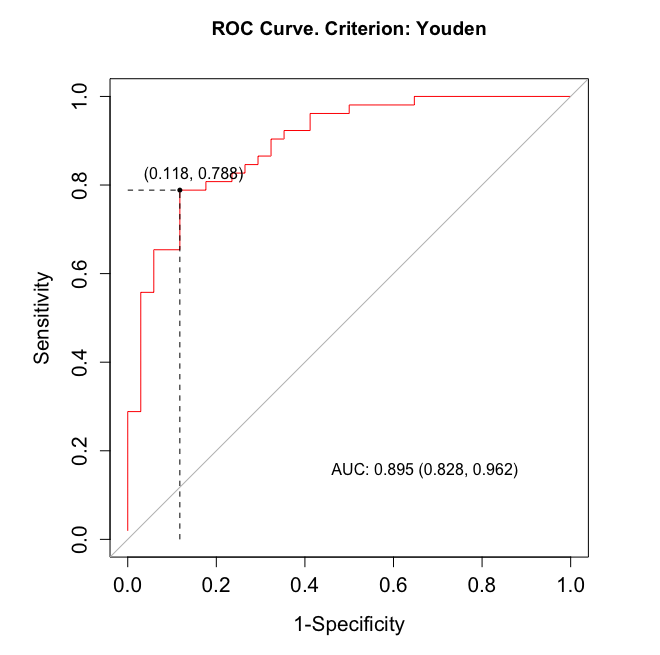

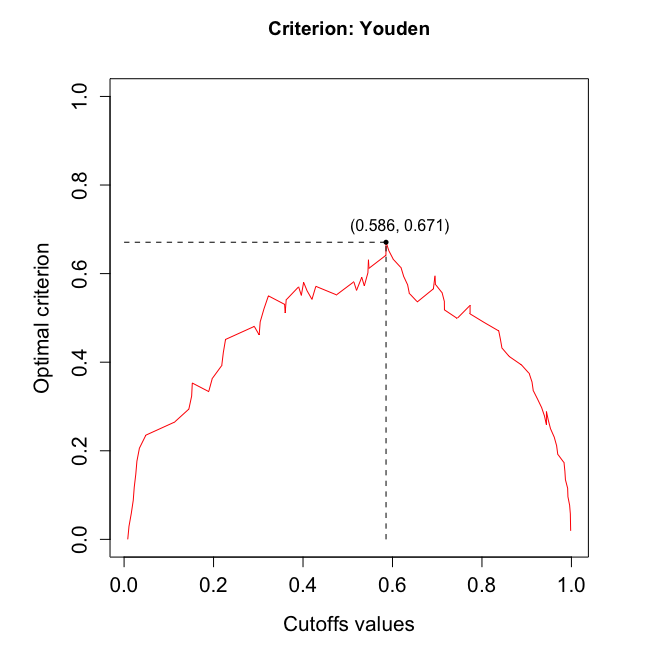

Supplement: Supplementary file 5 [file DataSheet_5.doc]
