# Supplementary material for: A Clinical Semantic and Radiomics Nomogram for Predicting Brain Invasion in WHO Grade II Meningioma Based on Tumor and Tumor-to-Brain Interface Features
Source: Front Oncol. 2021 Oct 22;11:752158. doi: 10.3389/fonc.2021.752158 (PMC8570084; doi:10.3389/fonc.2021.752158)
Supplement: Supplementary file 6 [file DataSheet_6.docx]

**Table S1 - AUC differences and P values of all models**

| **Comparison between models** | | **P value (AUC, Delong test）** | |
| --- | --- | --- | --- |
| **Model 1** | **Model 2** | **Training set (n=198)** | **Test set (n=86)** |
| CSRN | TRM | 0.0004548** <0.01 | 0.004364**<0.01 |
| CSRN | TbRM | 0.03929* <0.05 | 0.03983* <0.05 |
| CSRN | CSM | 0.03775* <0.05 | 0.03349* <0.05 |
| CSRN | TCTbRM | 0.07251 | 0.04661* <0.05 |
| TRM | TbRM | 0.01979* <0.05 | 0.2301 |
| TRM | CSM | 0.1487 | 0.4419 |
| TRM | TCTbRM | 0.0256* <0.05 | 0.1229 |
| TbRM | CSM | 0.9716 | 0.9068 |
| TbRM | TCTbRM | 0.4496 | 0.4929 |
| CSM | TCTbRM | 0.432 | 0.4301 |

**Table S2- ACC differences and P values of all models**

| **Comparison between models** | | **P value (ACC, z test）** | |
| --- | --- | --- | --- |
| **Model 1** | **Model 2** | **Training set (n=198)** | **Test set (n=86)** |
| CSRN | TRM | 9.234848e-05* *<0.01 | 0.05089511 |
| CSRN | TbRM | 0.05314676 | 0.08680513 |
| CSRN | CSM | 0.2836009 | 0.448701 |
| CSRN | TCTbRM | 0.2836009 | 0.6985458 |
| TRM | TbRM | 0.05667621 | 0.8681377 |
| TRM | CSM | 0.005919297* *<0.01 | 0.3045974 |
| TRM | TCTbRM | 0.005919297* *<0.01 | 0.1651793 |
| TbRM | CSM | 0.4588285 | 0.4877257 |
| TbRM | TCTbRM | 0.4588285 | 0.2907211 |
| CSM | TCTbRM | 1.0 | 0.8541788 |

**Table S3- SEN differences and P values of all models**

| **Comparison between models** | | **P value (SEN, z test）** | |
| --- | --- | --- | --- |
| **Model 1** | **Model 2** | **Training set (n=198)** | **Test set (n=86)** |
| CSRN | TRM | 1.259672e-05* *<0.01 | 0.1297498 |
| CSRN | TbRM | 0.2060485 | 0.09908787 |
| CSRN | CSM | 0.6727232 | 0.99 |
| CSRN | TCTbRM | 0.8355537 | 0.999 |
| TRM | TbRM | 0.002436735* *<0.01 | 1 |
| TRM | CSM | 9.174011e-07* *<0.01 | 0.1983556 |
| TRM | TCTbRM | 2.29905e-06* *<0.01 | 0.1983556 |
| TbRM | CSM | 0.06104469 | 0.1983556 |
| TbRM | TCTbRM | 0.09593966 | 0.1983556 |
| CSM | TCTbRM | 1.0 | 1 |

**Table S4- SPE differences and P values of all models**

| **Comparison between models** | | **P value (SPE, z test）** | |
| --- | --- | --- | --- |
| **Model 1** | **Model 2** | **Training set (n=198)** | **Test set (n=86)** |
| CSRN | TRM | 0.6009391 | 0.339925 |
| CSRN | TbRM | 0.1907407 | 0.7320459 |
| CSRN | CSM | 0.04655747* <0.05 | 0.339925 |
| CSRN | TCTbRM | 0.06827635 | 0.7320459 |
| TRM | TbRM | 0.5599064 | 0.7642444 |
| TRM | CSM | 0.2060308 | 1.0 |
| TRM | TCTbRM | 0.2725154 | 0.7642444 |
| TbRM | CSM | 0.6056633 | 0.7642444 |
| TbRM | TCTbRM | 0.7293298 | 1.0 |
| CSM | TCTbRM | 1.0 | 0.7642444 |
